# Supplementary material for: Aminoacyl chain translocation catalysed by a type II thioesterase domain in an unusual non-ribosomal peptide synthetase
Source: Nat Commun. 2022 Jan 10;13:62. doi: 10.1038/s41467-021-27512-0 (PMC8748450; doi:10.1038/s41467-021-27512-0)
Supplement: Supplementary file 1 — Supplementary information [file 41467_2021_27512_MOESM1_ESM.pdf]

Supplementary Information

Aminoacyl Chain Translocation Catalysed by a Type II Thioesterase Domain in an Unusual Non-Ribosomal Peptide Synthetase.

Shan Wang[1&], William D. G. Brittain[2&], Qian Zhang[3&], Zhou Lu[1], Ming Him Tong[1], Kewen Wu[1], Kwaku Kyeremeh[4], Matthew Jenner[5,6*], Yi Yu[3*], Steven L Cobb[2*], Hai Deng[1*]

1. Department of Chemistry, University of Aberdeen, Aberdeen, AB24 3UE, UK
2. Department of Chemistry, Durham University, Science Site, Durham, DH1 3LE, UK
3. Key Laboratory of Combinatorial Biosynthesis and Drug Discovery (MOE) and Hubei Province Engineering and Technology Research Centre for Fluorinated Pharmaceuticals, School of Pharmaceutical Sciences, Wuhan University, Wuhan 430071, China
4. Department of Chemistry, University of Ghana, P.O. Box LG56, Legon-Accra, Ghana
5. Department of Chemistry, University of Warwick, Coventry, CV4 7AL, UK.
6. Warwick Integrative Synthetic Biology (WISB) Centre, University of Warwick, Coventry, CV4 7AL, UK

[&] these authors contributed equally to the work

Supplementary Methods

Synthesis of methyl O-(*tert*-butyl)-*N*-(3-methylbutanoyl)-L-threoninate

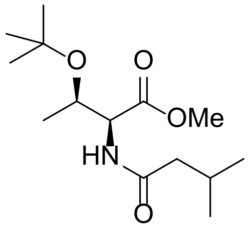

To a solution of H-Thr(O*t*Bu)-OMe·HCl (0.30 g, 1.33 mmol) and TEA (0.39 mL, 2.79 mmol) in DCM (20 mL) was added isovaleryl chloride (0.18 mL, 1.46 mmol) and the reaction mixture was stirred for 16 h at rt. After this period the reaction mixture was concentrated, and the residue purified directly by flash column chromatography (100% hexane to 100% EtOAc). This gave the desired product as a clear oil in a 62% yield (0.224 g).

^{1}H NMR (400 MHz, $CDCl_3$) δ 6.20 (brd, *J* = 9.3, 1H), 4.56 (dd, *J* = 9.3, 1.8, 1H), 4.25 (qd, *J* = 6.3, 1.8, 1H), 3.72 (s, 3H), 2.35 – 1.99 (m, 3H), 1.20 (d, *J* = 6.3, 3H), 1.13 (s, 9H), 1.01 (dd, *J* = 6.3, 3.6, 6H).

^{13}C NMR (101 MHz, $CDCl_3$) δ 173.03, 171.51, 74.10, 67.37, 57.56, 52.14, 45.96, 28.33, 26.19, 22.50, 22.44, 21.06.

HRMS ESI$^+$ Calculated for $[M+H]^+$ $C_{14}H_{28}NO_4^+$ = 274.2018 Found = 274.2005

Synthesis of O-(*tert*-butyl)-*N*-(3-methylbutanoyl)-L-threonine **12a**

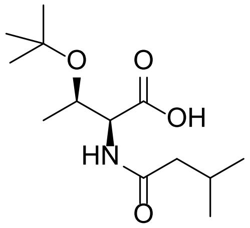

To a solution of methyl O-(*tert*-butyl)-*N*-(3-methylbutanoyl)-L-threoninate (0.880 g, 3.22 mmol) in a THF:H_2O (2:1) (10 mL) was added LiOH·H_2O (0.405 g, 9.66 mmol) and the reaction was stirred vigorously for 3 h at rt. After this time the reaction mixture was acidified to pH 3 with 1M HCL(aq) and immediately extracted with EtOAc (3 x 25 mL). The combined organic fractions were dried over $MgSO_4$ and concentrated under reduced pressure to yield the desired compound as a clear oil in 92% yield (0.768 g).

^{1}H NMR (400 MHz, $CDCl_3$) δ 6.39 (brd, *J* = 5.8, 1H), 4.51 (dd, *J* = 5.8, 4.2, 1H), 4.41 (qd, *J* = 6.4, 4.2, 1H), 2.21 – 2.08 (m, 3H), 1.33 (s, 9H), 1.13 (d, *J* = 6.4, 3H), 1.04 – 0.93 (m, 6H).

^{13}C NMR (101 MHz, $CDCl_3$) δ 173.05, 66.08, 60.44, 57.05, 45.65, 28.04, 26.15, 22.47, 22.44, 14.21.

HRMS ESI$^+$ Calculated for $[M+H]^+$ $C_{13}H_{25}NO_4^+$ = 260.1862 Found = 260.1851

Synthesis of pantetheine from pantothenic acid **13**

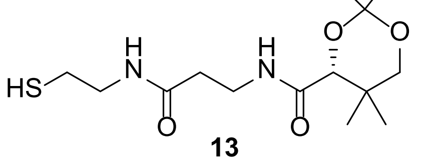

13

The thiol **13** was prepared according to the previous procedure.[1] Briefly, to a solution of protected pantothenic acid (200 mg, 0.8 mmol) in CH_2Cl_2 (4 mL) was added cysteamine

hydrochloride (200 mg, 1.6 mmol), PyBOP (605 mg, 1.2 mmol) and diisopropylethylamine (0.6 mL, 3.8 mmol). The reaction was allowed to stir for overnight under the nitrogen protection. The solvent was removed and the residue was subjected to flash column chromatography to afford **13**.

^{1}H-NMR (400 MHz, $CDCl_3$): δ 7.01 (bs, 1H, NH), 6.34 (bs, 1H, NH), 4.07 (s, 1H), 3.66 (d, J = 11.8 Hz, 1H), 3.61-3.50 (m, 2H), 3.49-3.37 (m, 2H), 3.26 (d, J = 11.7 Hz, 1H), 2.69-2.60 (m, 2H), 2.47 (t, J =6.3 Hz, 2H), 1.42 (s, 3H), 1.41 (s, 3H), 1.37 (t, 1H), 1.03 (s, 3H), 0.97 (s, 3H);

^{13}C-NMR (150 MHz, $CDCl_3$): δ 172.0, 170.6, 99.6, 77.6, 71.9, 43.6, 36.4, 35.3, 33.4, 29.9, 26.8, 22.6, 19.4, 19.2; HRMS (ESI) *m/z* calculated for $C_{14}H_{26}N_2O_4SNa$ ($[M+Na]^+$) 341.1505, found 341.1490.

Synthesis of *S*-(2-acetamidoethyl) (2*S*,3*R*)-3-(tert-butoxy)-2-(3-methylbutanamido)butanethioate **14**

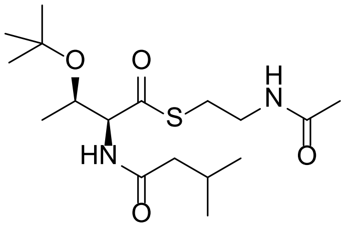

To a solution of *O*-(*tert*-butyl)-*N*-(3-methylbutanoyl)-L-threonine (0.663 g, 2.56 mmol), DIPEA (0.660 g, 5.12 mmol) and EDC.HCl (0.540 g, 2.82 mmol) in DCM (30 mL) was added N-acetylcysteamine (0.305 g, 2.56 mmol) and the reaction was stirred overnight at rt. The reaction mixture was then concentrated under reduced pressure the residue was then directly purified by flash column chromatography (100% DCM to 97% DCM 3% MeOH). This gave the desired product as a clear oil which slowly solidified to a white solid in 54% yield (0.497 g).

^{1}H NMR (400 MHz, $CDCl_3$) δ 6.09 – 5.93 (brm, 2H), 4.73 (dd, *J* = 7.6, 4.4, 1H), 4.10 (qd, *J* = 6.5, 4.4, 1H), 3.51 – 3.37 (m, 2H), 3.05 (appt, *J* = 5.9, 2H), 2.23 – 2.12 (m, 3H), 1.97 (s, 3H), 1.21 (s, 9H), 1.15 (d, *J* = 6.5, 3H), 1.05 – 0.97 (m, 6H).

^{13}C NMR (101 MHz, $CDCl_3$) δ 199.47, 173.25, 170.49, 74.89, 67.04, 64.44, 45.96, 39.15, 28.53, 28.20, 26.25, 23.19, 22.57, 22.50, 18.51.

HRMS ESI$^+$ Calculated for $[M+H]^+$ $C_{17}H_{33}N_2O_4S^+$ = 361.2161 Found = 361.2137

Addition of TFA facilitated the conversion of protected **14** to IV-Thr-Pro-SNAC **14a** prior to the biochemical reactions.

Supplementary table

Supplementary Table 1. Primers used in this study.

Primer	Sequence	Use
LgnA_EI_for	TAAGAATTCATGCCTGTCACACTTTTGTGC ATCCCGTAT	Amplification of *lgnA*
LgnA_Hd_rev	ATAAAGCTTCTCAGGCGGCGGAACGGGC GAACAC	
LgnB_for	GTCGCGGATCCGAATTCATGACCGCCACG ATTCCC	Amplification of *lgnB*
LgnB _rev	AGTGCGGCCGCAAGCTTCCGTGATCGTTG TGTTCAGG	
LgnD_CHisfor	AGAAGGAGATATACATATGATGCACGCTC GCCCGCTGTA	Amplification of *lgnD*
LgnD_CHisrev	TGGTGGTGGTGCTCGAGTCGGGCACCGC CGGCGGCCTCGT	
LgnD_for	GTCGCGGATCCGAATTCATGCACGCTCGC CCGCTGTA	Amplification of *lgnD-*C_1T_1 or *lgnD-*$C_1T_1C_2$
LgnD_CT1rev	AGTGCGGCCGCAAGCTTGTCCTCGAAGG CGCTGAGCC	Amplification of *lgnD-*C_1T_1
LgnD_C2rev	AGTGCGGCCGCAAGCTTGTCCACCGTCTC CTGGGGGCGTT	Amplification of *lgnD-*$C_1T_1C_2$
LgnA_28afor	GTCGCGGATCCGAATTCATGCCTGTCACA CTTTTGT	
LgnA_28arev	TCGAGTGCGGCCGCAAGCTTCTCAG	Construction of LgnAS77A variant
LgnA_S77toArev	AGCGCGCCGAACGCGTGTCCGAAGAG	
LgnA_S77toAfor	GACACGCGTTCGGCGCGCTCGTCG	
LgnD_T1_StoAfor	CGATGCGATCACGATGCTGAAGAT	Construction of C_1-T_1(S492A) and C_1-T_1(S492A)-C_2 variants
LgnD_T1_StoArev	TCAGCATCGTGATCGCATCG	
28a-LgnD_C1-F	CTGGTGCCGCGCGGCAGCCATATGCACG CTCGCCCGCTGTACCCGCTC	Amplification of *lgnD-*C_1

28a-LgnD_C1-R	GTGCTCGAGTGCGGCCGCAAGCTTTCACAGCGCGGGCAGCGTGCCGACCG	
28a-LgnD_T1-F	CTGGTGCCGCGCGGCAGCCATATGGCGCCGCACACCGGTGCACAGCCG	Amplification of *lgnD-T_1*
28a-LgnD_T1-R	GTGCTCGAGTGCGGCCGCAAGCTTTCACGCCCGTGCGGCCAGCTCCGCC	

Restriction sites are underlined.

Supplementary Table 2. Amino acid sequences of LgnB-A_1-T_0 and truncated LgnD-C_1-T_1 for intact protein MS analysis.

Protein	Sequence
LgnA (Accession no: AIZ66876.1)	MGSSHHHHHHSSGLVPRGSHMASMTGGQQMGRGSEFMPVTLLCIPY AGAGASLFRRWKRHPFQHMDVAAVQLPGREELFADGPCTSMSELVDL CAGHIRELPQDAPFALFGHSFGALVAYETAQRLAAEGLRLPERLIVSGA AAPWLPRPVTDADSLSDDQFVARVRDVVGYDHPALHDAELRGLLLPSL RADLSISDRYAPGSTDPLPVPLTVLRGSDDRLVSRQDVELWAKAASQP TELIELPGDHMYFSLDPKPLLAELDAVFARSAA
LgnB-A_1-T_0 (Accession no: AIZ66877.1)	MGSSHHHHHHSSGLVPRGSHMASMTGGQQMGRGSEFMTATIPGLFG RQARRIPDRPAVTCGEETITYAALDARSTGLAAALREHGVRPGDRVGV CLARGIDLVVALLAVLKAGAAYVPLDPGYPPQRLAFVAEDTGLTVAVTDT QVPGLTSVPVTAAAGRPAGALPLPEPDAVAYVIHTSGSTGRPKGVLVPH RNVAALLAATAGEFGFGEDDVWTLFHSFAFDYSVWEIWGCLLTGGRLV VVPHWTARDPEAFHALLAAEGVTVLSQTPSAFTPLLRTSGFGNGELRV RLLVFGGEPLDTRVLVPWFERYPDARVENMYGITETTVHCTRRTLTLQD ARQGIRSVGRALPGWALYVLDEQGRQAGPGVPGEIHVGGAGVADGYL NRPELTAQRFLPDTITGGGGLLYRSGDRGRVLENGELEHLGRLDDQVKI RGHRIELGEIRGALLEDPHVRAAAAVVRDTADPANACVDAYVVTDRPD GVPALRERLAQQLPAYLVPATITAVDAFPLTPNGKIDTDRLPAPRTHGAA VPVETEAADPVTAQLTGIWQRLLEEPVGPEDNFFELGGNSLLFARLATEI RESGLGHVSLRDLYLTATLQDMARLIRSRADAA
LgnD-C_1-T_1. (Accession no: AIZ66879.1)	MGSSHHHHHHSSGLVPRGSHMASMTGGQQMGRGSEFMHARPLYPLS AHQRDIWFSDALMPESPQYSIVLREKFSGDLDLEALKAAVEYVLAHNDA FRIRIDSADGEPHQWLEPGEPELAVLDFRAESDPEAACLAWADERRTV RLPVPGERLYDAVLLRASDTKVYFQLTAHHLISDGWSLSETRKQVRDRY TETVRGEAAAGPAVPSFVDCIDADTRYRASESFAQDRQFLASALSGAE PRLFARKKPTAPLGHRTSFVIEGKVIDRILANGNSPFSVIAAAFGTCLARA HQADEVTLGVAMLNRHSAEERQALGVFANVLPLRVAADGSRSMSEVA EQVRMSVRGLQEHQRLPLADVLRDWRSSGRSGALFDVTISYLHFPEPP SVPGLSVDTHMALELLDGNALAIVVHAGEGADEVRVDLVYAADVFDDDF PMAEVVQAVQALIRQGVDLPDAPVGTLPALAGVRTAPHTGAQPAEERL PESGDSGDGTPRTTMEAELAAVWAEVLDLPSVGIHDNYFVLGGDSITM LKIRAGGERRGIRFSVTDIITGPTVAELAARAVLHAEDAPETPAVAPLELV PAVDRGRLSAFEDKLAAALEHHHHHH

Supplementary Table 3. Calculated and measured mass values for species detected using intact protein mass spectrometry.

Species	Calculated Mass / Da	Measured Mass / Da
holo-LgnB A_1-T_0	65815	65813
L-Thr-LgnB A_1-T_0	65916	65913
IV-Thr-LgnB A_1-T_0	66600	65998
holo-LgnD-C_1-T_1	66188	66185
L-Thr-LgnD-C_1-T_1	66289	66286
IV-Thr-LgnD-C_1-T_1	66373	66372
LgnA TE_{II}	29286	29284
L-Thr-LgnA TE_{II}	29387	29387

Supplementary Table 4. Protein information included in the phylogenetic tree analysis in Fig. 6d.

Protein	Accession	Organism
NocB_TE	AAT09805.1	*Nocardia uniformis* subsp. *tsuyamanensis*
TycC_TE	AAC45930.1	*Brevibacillus brevis*
Psy_TE	WP_064118616.1	*Pseudomonas fluorescens*
Srf_TE	1JMK_C	*Bacillus subtilis*
TdiA_TE	ABU51602.1	*Aspergillus nidulans*
GrsB_TE	CAA43838.1	*Brevibacillus brevis*
Fen_TE	2CBG_A	*Bacillus subtilis*
EntF_TE	CDQ53511.1	*Klebsiella pneumoniae*
TioS_TE	CAJ34375.1	*Micromonospora* sp. ML1
CndF_TE	CAQ43084.1	*Chondromyces crocatus*
LpiB_TE	AFV70301.1	*Pseudomonas fluorescens*
CTB1_TE	AAT69682.1	*Cercospora nicotianae*
NanE	CDQ55269.1	*Klebsiella pneumoniae*
MonCII	AAO65791.1	*Streptomyces cinnamonensis*
NigCII	ABC84462.1	*Streptomyces violaceusniger*
BarC	AAN32977.1	*Lyngbya majuscula*
KtzF	ABV56586.1	*Kutzneria* sp. 744
RedJ	CAA16185.1	*Streptomyces coelicolor* A3(2)
TioQ	CAJ34373.1	*Micromonospora* sp. ML1
Qui14	AET98912.1	*Streptomyces griseovariabilis* subsp. *bandungensis*
NikP2	CAC11138.1	*Streptomyces tendae*
Bhp	CAC48368.1	*Amycolatopsis balhimycina* DSM 5908
Thr4	CCF23460.1	*Streptomyces* sp.
ClbQ	BBW60695.1	*Escherichia coli*
TylO	AAA21345.1	*Streptomyces fradiae*
BorB	CAE45660.1	*Streptomyces parvulus*
RifR	AAG52991.1	*Amycolatopsis mediterranei* S699
CmaE	AAO58147.1	*Pseudomonas syringae* pv. tomato str. DC3000
SyrC	AAA85161.1	*Pseudomonas syringae* pv. *syringae*
CouN7	AAG29792.1	*Streptomyces rishiriensis*
CloN7	AAN65236.1	*Streptomyces roseochromogenus* subsp. *oscitans* DS 12.976
SpsS	WP_076971574.1	*Streptomyces sparsogenes*
RthD	WP_012998270.1	*Streptomyces scabiei*

A0A2N1EF60	A0A2N1EF60	*Pseudomonas fluorescens*
A0A2S6Y2U9	A0A2S6Y2U9	*Xanthomonas arboricola*
A0A158JM70	A0A158JM70	*Caballeronia choica*
A0A3T0VQM2	A0A3T0VQM2	*Hahella* sp. KA22
A0A6C1CFP1	A0A6C1CFP1	*Streptomyces albus*
Q79Z91	Q79Z91	*Streptomyces avermitilis*
WP_016335672.1	WP_016335672.1	*Amycolatopsis keratiniphila*
WP_120720255.1	WP_120720255.1	*Streptomyces hundungensis*
WP_090051576.1	WP_090051576.1	*Lechevalieria fradiae*
WS5	QBA57731.1	*Streptomyces sp. SNM55*
WS20	QBA57738.1	*Streptomyces sp. SNM55*

Supplementary Figures

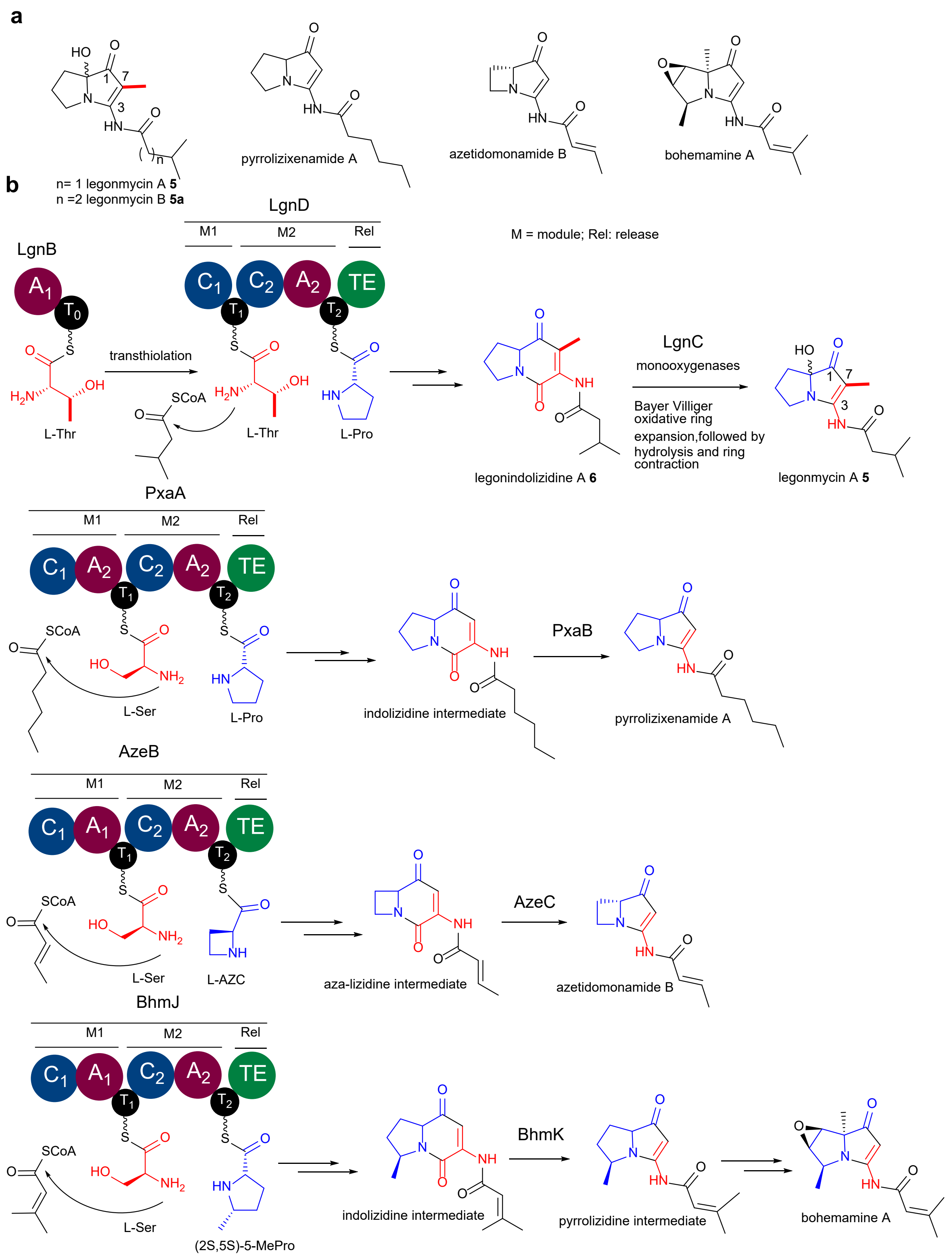

Supplementary Fig. 1. a. Structural comparison of representative bacterial pyrrolizidine alkaloids (PAs), indicating that legonmycins **5** contain an extra methyl group (red bald bond) at C-7 position. **b**. Comparison of the architecture of NRPS enzymes responsible for the

indolizidine intermediates in the biosynthetic pathway of bacterial PAs, indicating that most of PA pathways consist of canonical multidomain NRPS.

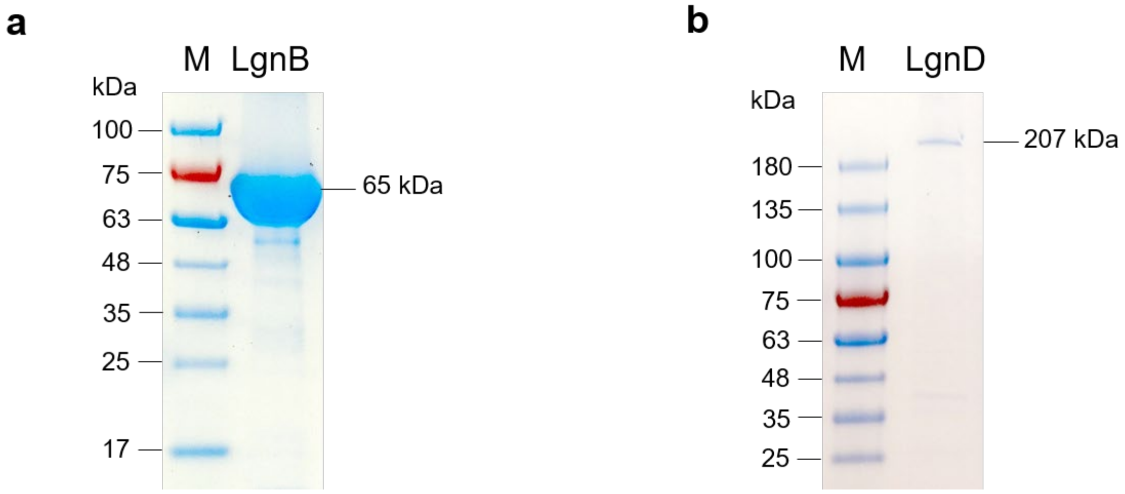

Supplementary Fig. 2. SDS-PAGE analysis of recombinant N-His$_6$–tagged LgnB (**a**) and C-His$_6$–tagged LgnD (**b**). All experiments were repeated independently with similar results for three times.

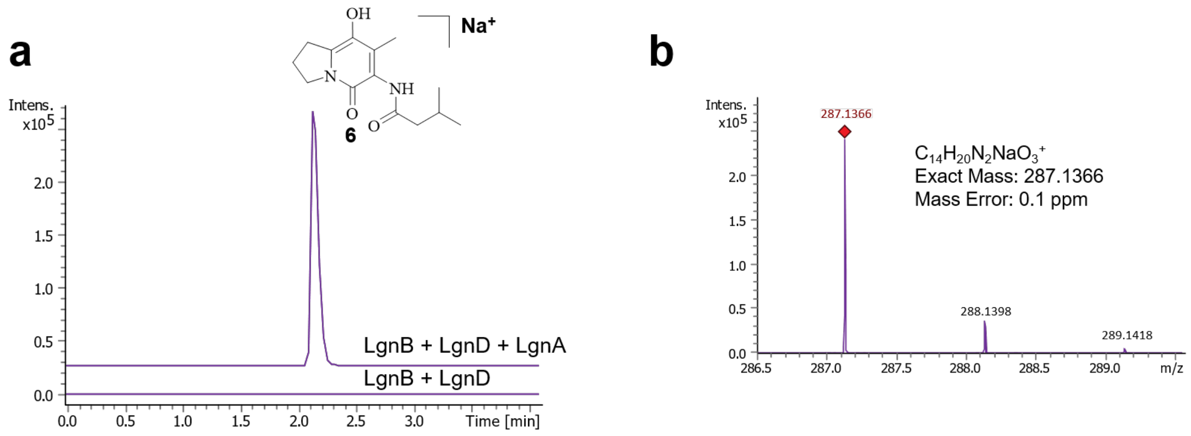

Supplementary Fig. 3. UHPLC-HR-ESI-MS analysis of legonindolizidine A **6** generated in the one-pot reconstitution of LgnA, LgnB and LgnD with substrates and cofactors. **a**. Extracted ion chromatograms (EICs) of the presence of **6** (m/z 287.1366 $[M+Na]^+$) (top) when LgnA was added, and the absence of **6** (bottom) when LgnA was omitted. **b**. HR-MS spectrum of **6**.

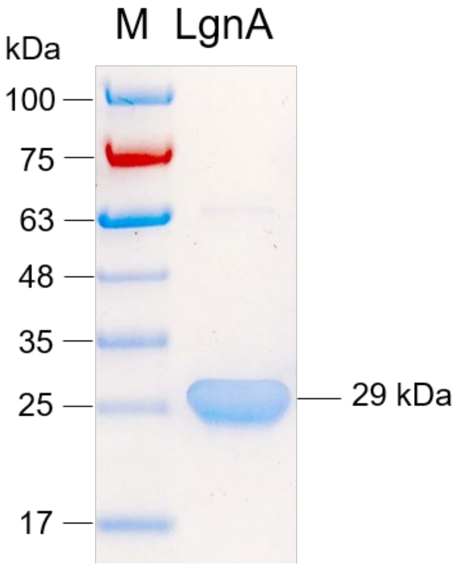

Supplementary Fig. 4. SDS-PAGE analysis of recombinant N-His$_6$–tagged LgnA. All experiments were repeated independently with similar results for three times.

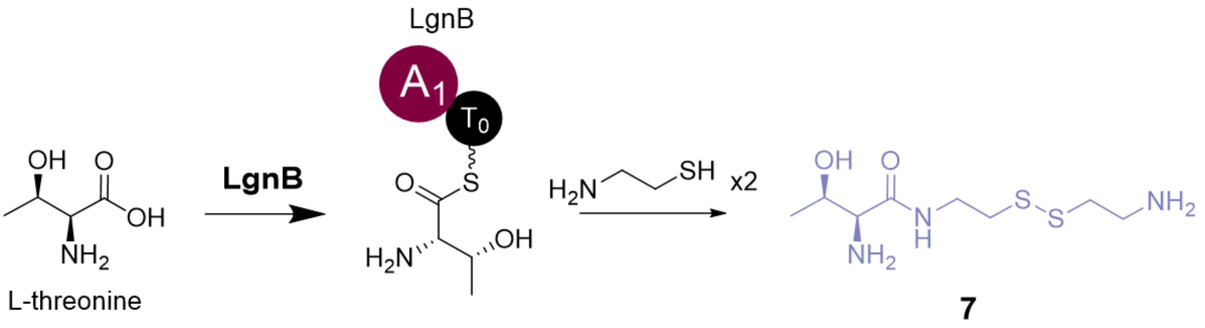

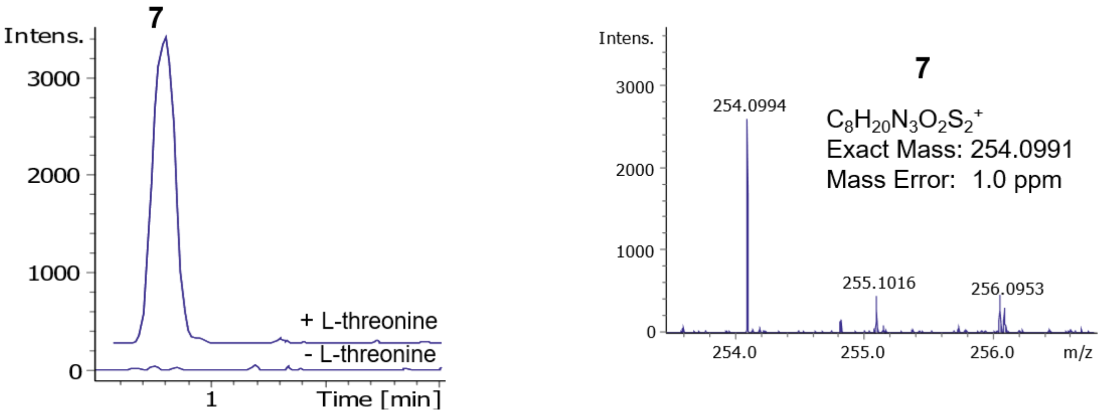

Supplementary Fig. 5. LC-HRMS analysis of chemical capturing of expected NRPS-bound intermediates when the assay was performed in one-pot reaction of LgnB with substrates. Extracted ion chromatograms (EICs) and mass spectrum of the cystamine-adducted Thr **7** (*m/z* 254.0991 $[M+H]^+$) in the chemical capture assay of LgnB containing A_1-T_0 domains with or without L-Thr and cofactors.

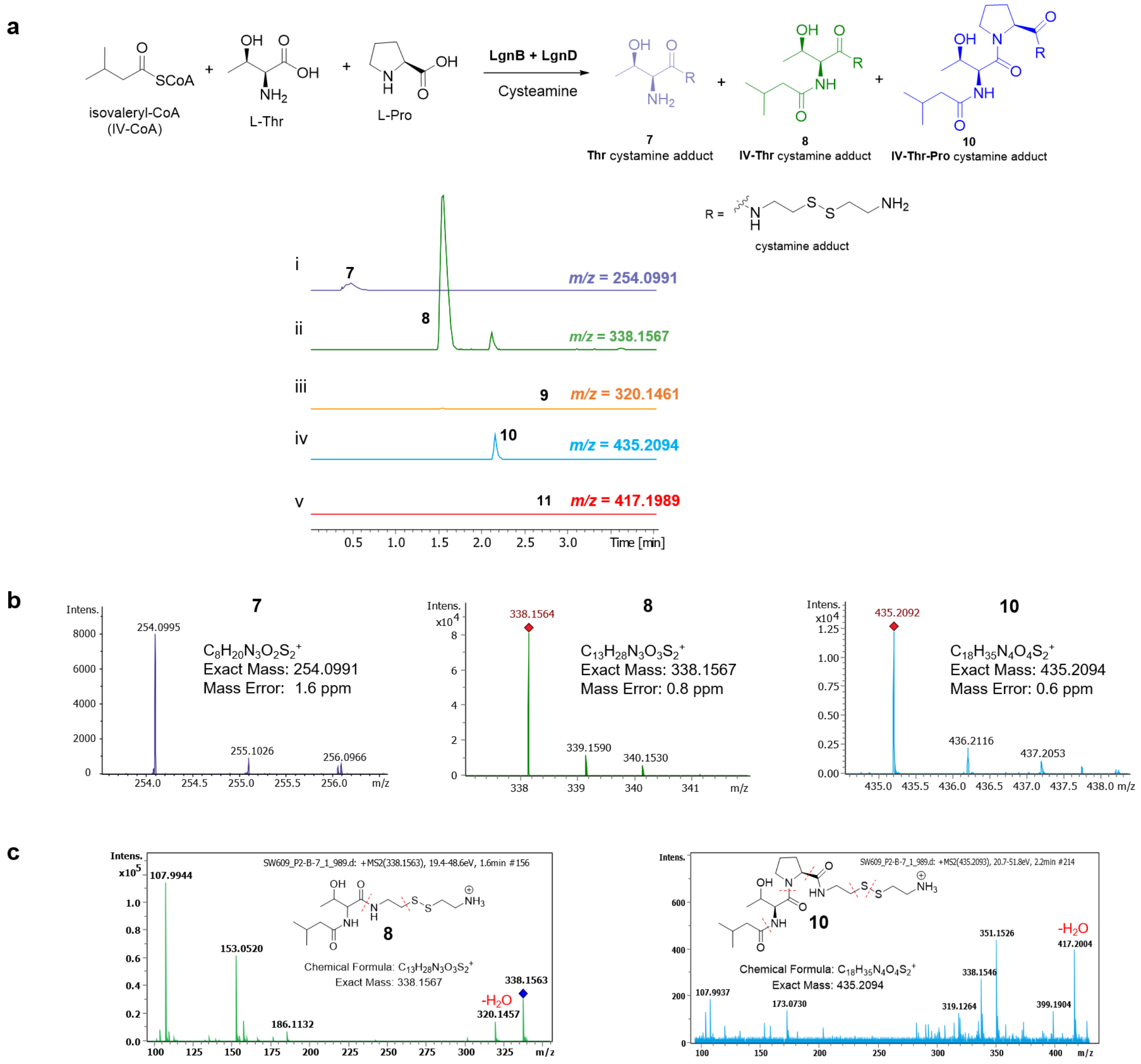

Supplementary Fig. 6. LC-HRMS analysis of chemical capturing of expected NRPS-bound intermediates when the assay was performed in one-pot reaction of LgnB and LgnD with substrates and cofactors. **a** NRPS bound intermediates captured by cysteamine in the one-pot assay. **b** MS spectra of **7, 8** and **10**. **c** MS^2 fragmentation analysis of **8** and **10**.

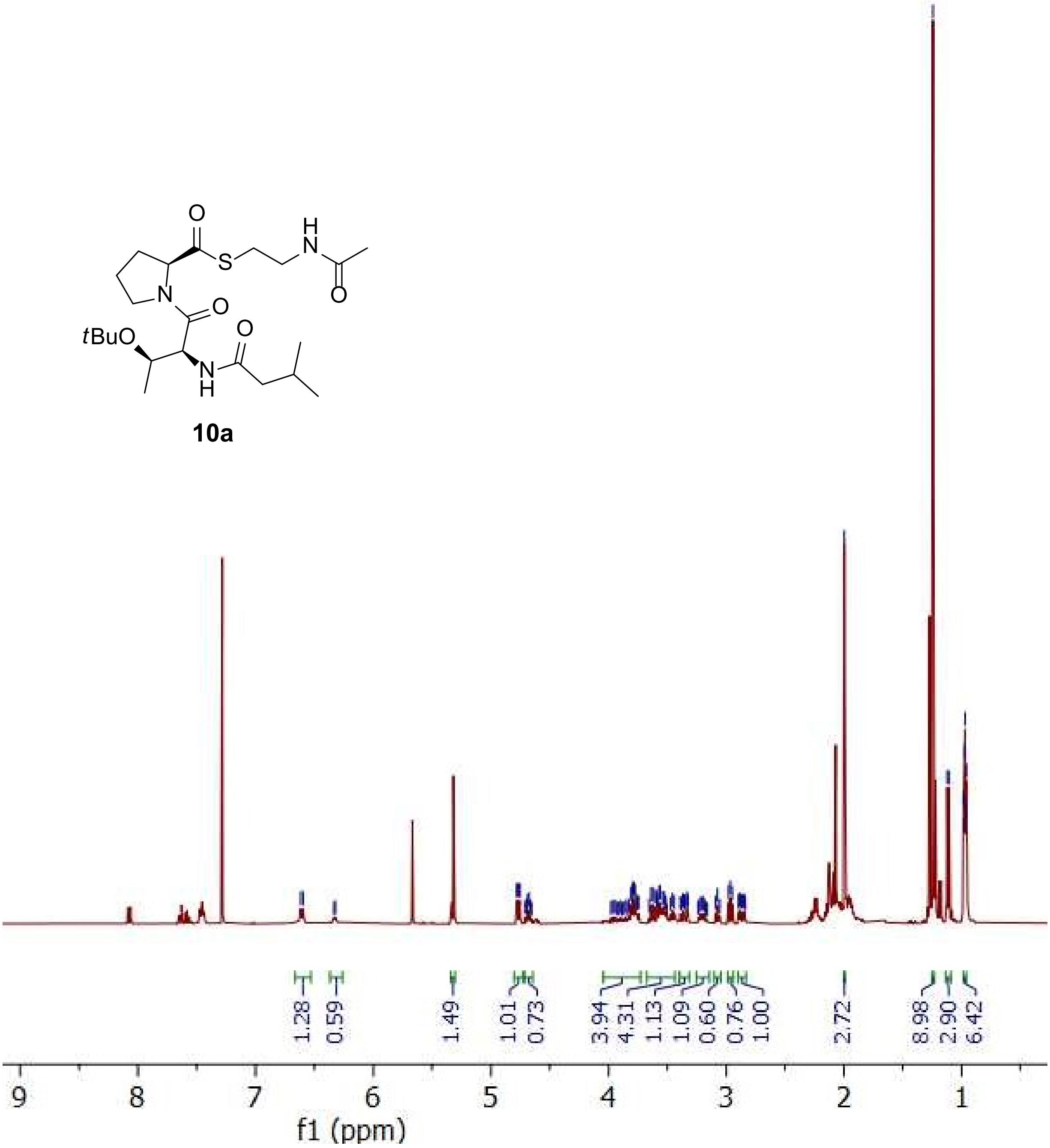

Supplementary Fig. 7. ^{1}H NMR spectrum of isovaleryl(IV)-O-*t*Bu-L-Thr- L-Pro-SNAC **10b** in $CDCl_3$ (400 MHz, 28°C)

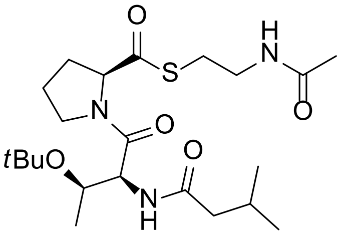

10b

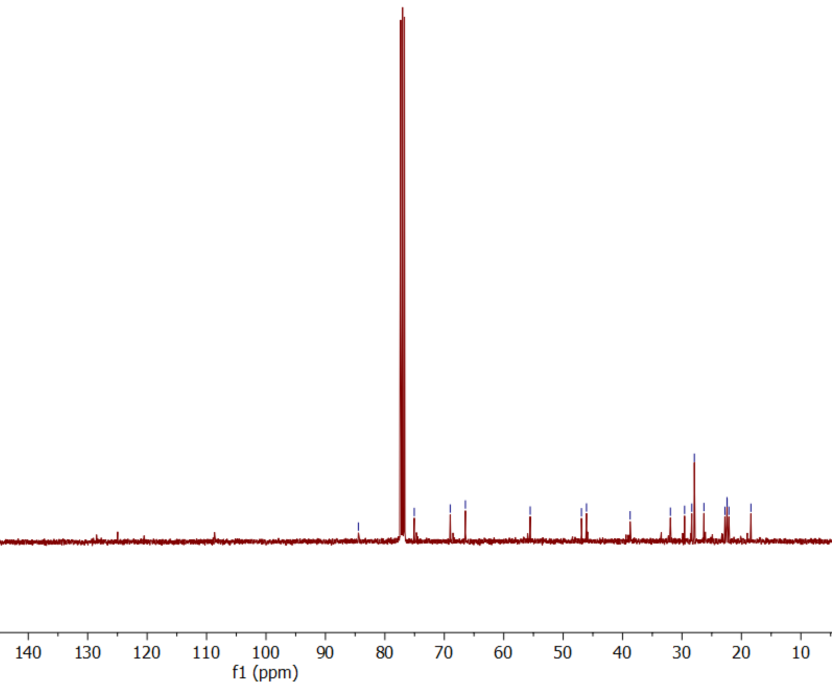

Supplementary Fig. 8. ^{13}C NMR spectrum of IV-O-*t*Bu-L-Thr-L-Pro-SNAC **10b** in $CDCl_3$ (150 MHz, 28°C)

Elemental Composition Report

Single Mass Analysis

Tolerance = 3.0 mDa / DBE: min = -1.5, max = 50.0
Element prediction: Off
Number of isotope peaks used for i-FIT = 3

Monoisotopic Mass, Even Electron Ions
2623 formula(e) evaluated with 4 results within limits (up to 500 best isotopic matches for each mass)
Elements Used:
C: 0-50 H: 0-50 N: 0-6 O: 0-9 S: 0-5 160Dy: 0-1

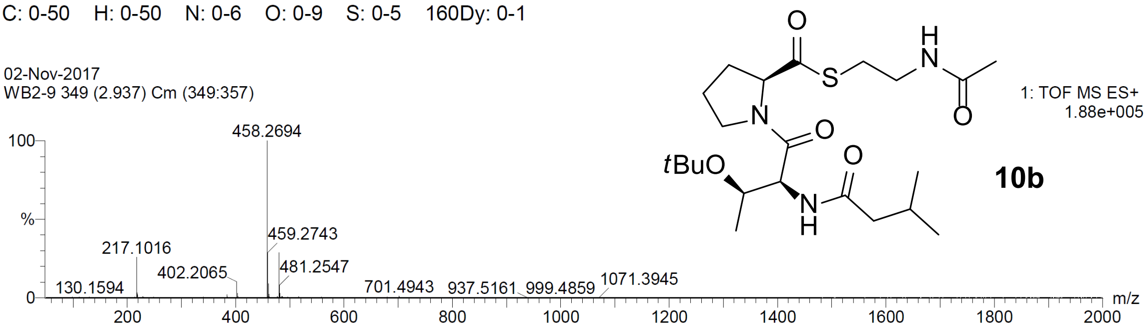

Minimum:				-1.5			
Maximum:		3.0	5.0	50.0			
Mass	Calc. Mass	mDa	PPM	DBE	i-FIT	i-FIT (Norm)	Formula
458.2694	458.2689	0.5	1.1	4.5	483.9	0.0	C22 H40 N3 O5 S
	458.2722	-2.8	-6.1	-0.5	492.9	8.9	C19 H44 N3 O5 S2
	458.2695	-0.1	-0.2	13.5	494.4	10.5	C30 H36 N O3
	458.2697	-0.3	-0.7	3.5	495.6	11.6	C23 H44 N3 S3

Supplementary Fig. 9. Elemental analysis of IV-O-*t*Bu- L-Thr- L-Pro-SNAC **10b**.

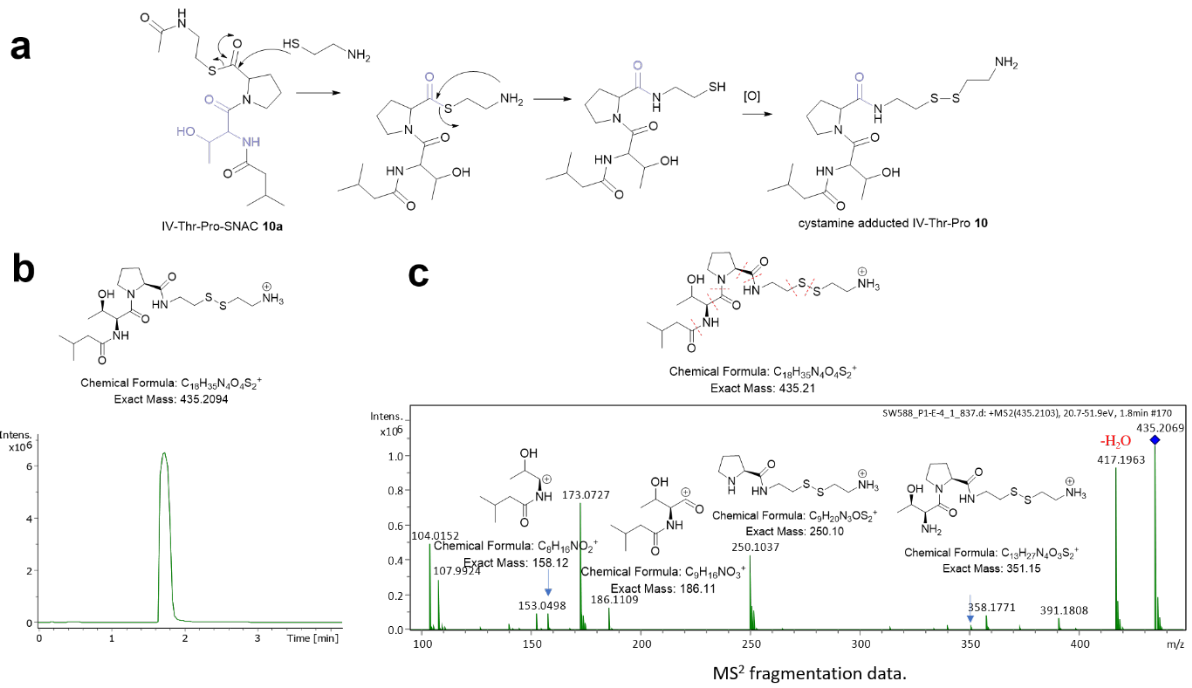

Supplementary Fig. 10. LC-MS analysis of chemically captured IV-Thr-Pro-SNAC in the presence of cysteamine. **a**. Proposed mechanism of chemical cleavage of thioester in the presence of cysteamines. **b**. LC-MS analysis of cystamine adducted product, IV-Thr-Pro cystamine adduct **10**, generated from the synthetic IV-Thr-Pro-SNAC **10a**. **c**. MS^2 fragmentation analysis of **10** from **10a** which has the identical MS^2 fragment pattern as the one generated from the one-pot biotransformation.

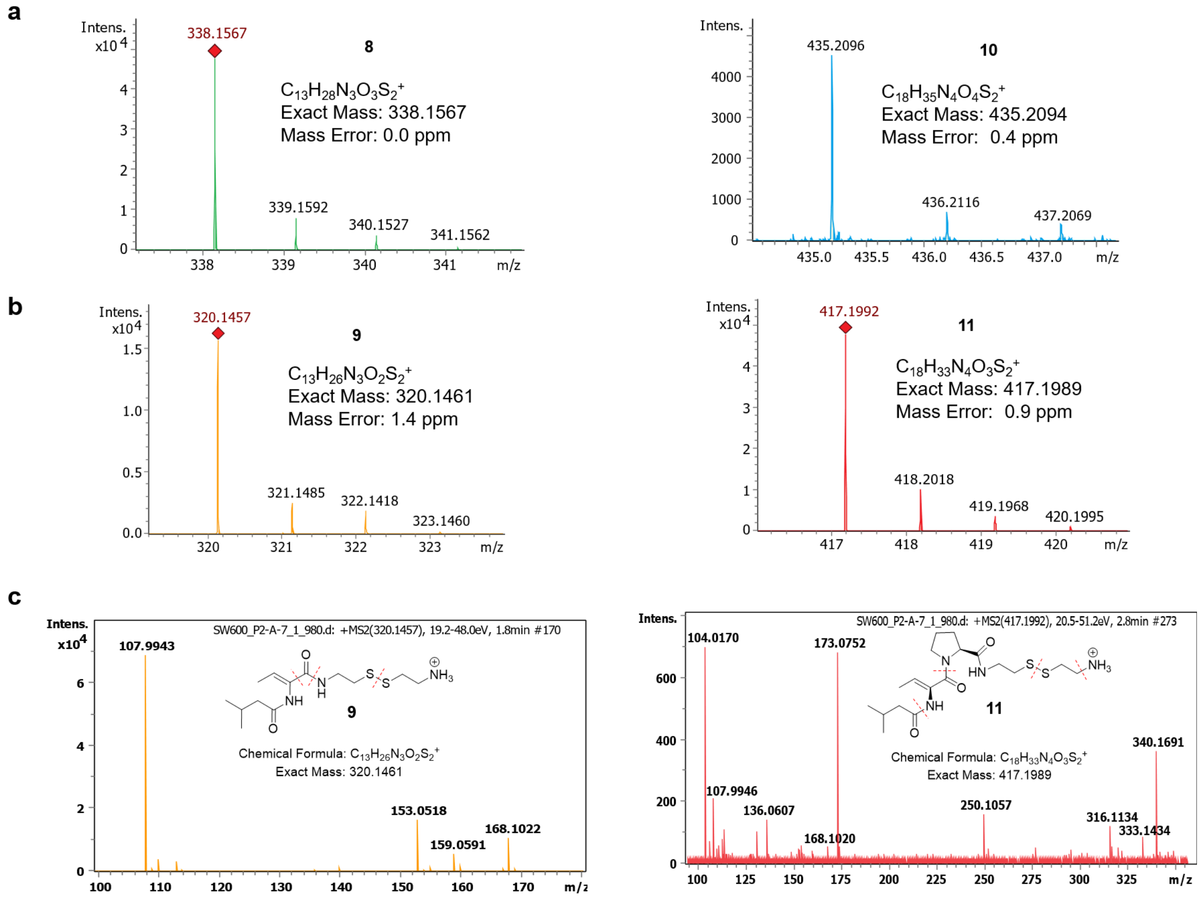

Supplementary Fig. 11. MS spectra and MS^2 fragmentation of cystamine-adducted compounds, **8-11** from the reaction mixture of the one-pot reconstitution of LgnA, LgnB and LgnD with the presence of cysteamine. (**a**) MS spectra of **8** (left) and **10** (right); (**b**) MS spectra of **9** (left) and **11** (right); (**c**) MS^2 fragmentation of **9** (left) and **11**(right).

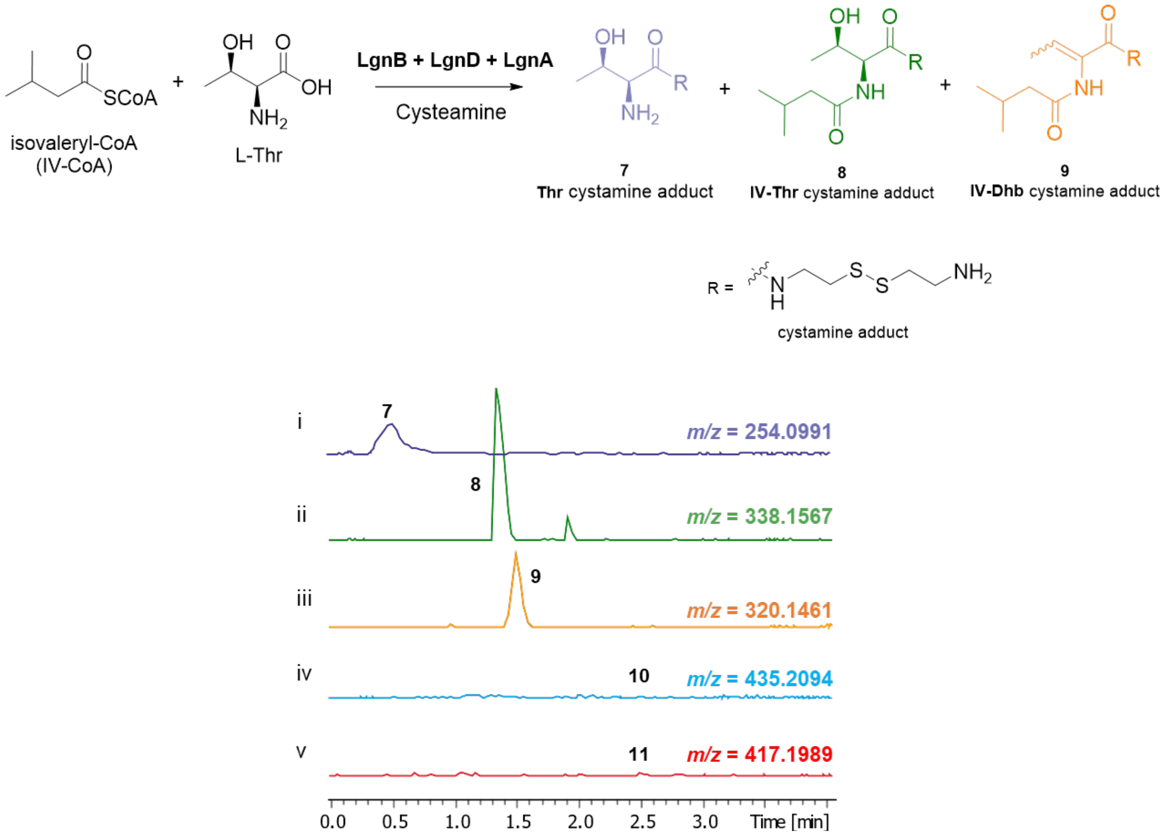

Supplementary Fig. 12. Extracted ion chromatograms (EICs) of cystamine adducted **7-9** from the reaction mixture of the assay of LgnB, LgnA and LgnD together with L-Thr, IV-CoA, and ATP but omitting Pro. All LC-MS traces were represented in the same scale of ion intensity (10^4).

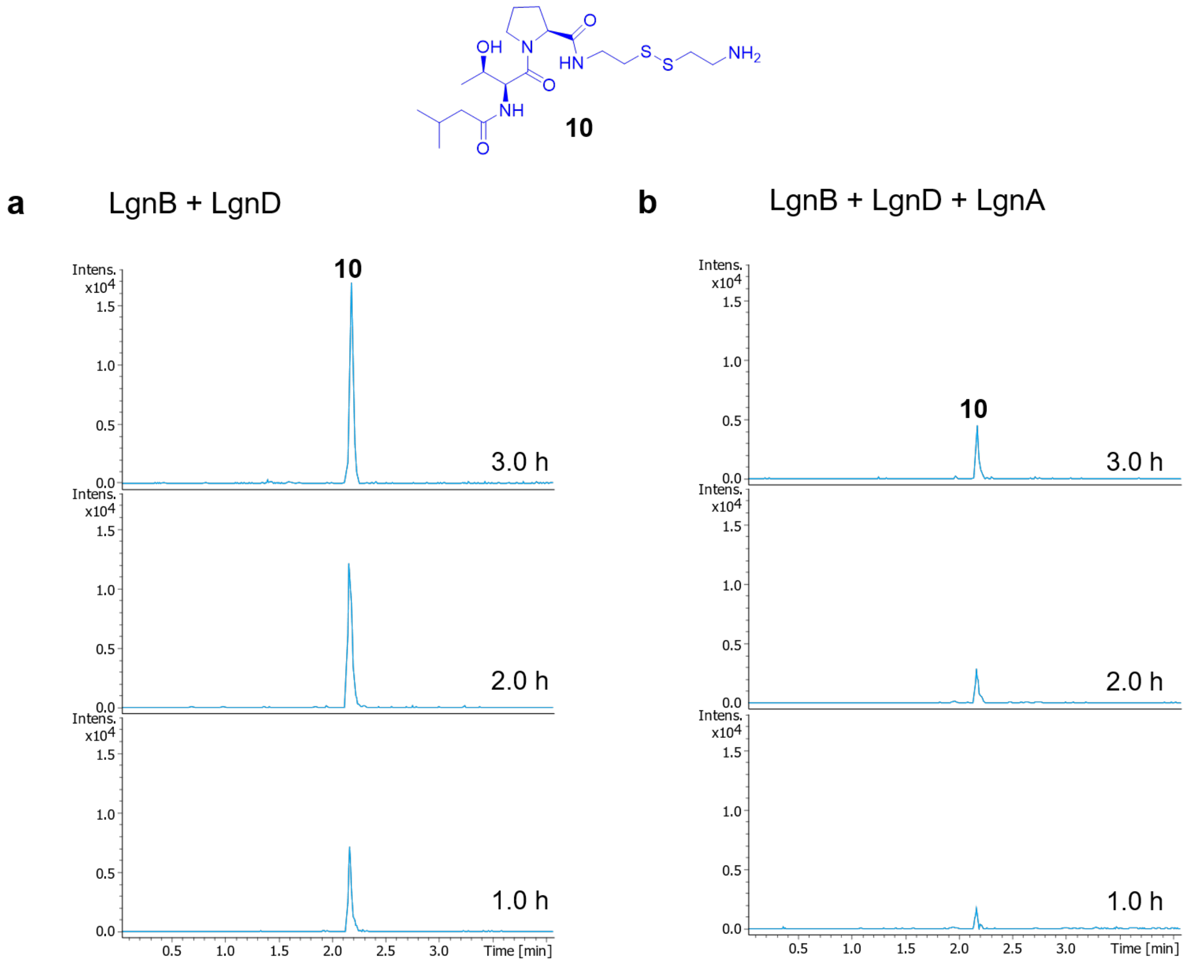

Supplementary Fig. 13. UHPLC-HR-ESI-MS analysis of chemical offloading of NRPS-bound intermediate, **10**. **a**. The time course of the presence of **10** in the presence of LgnB and LgnD. **b**. The time course of the presence of **10** in the presence of LgnB and LgnD with LgnA.

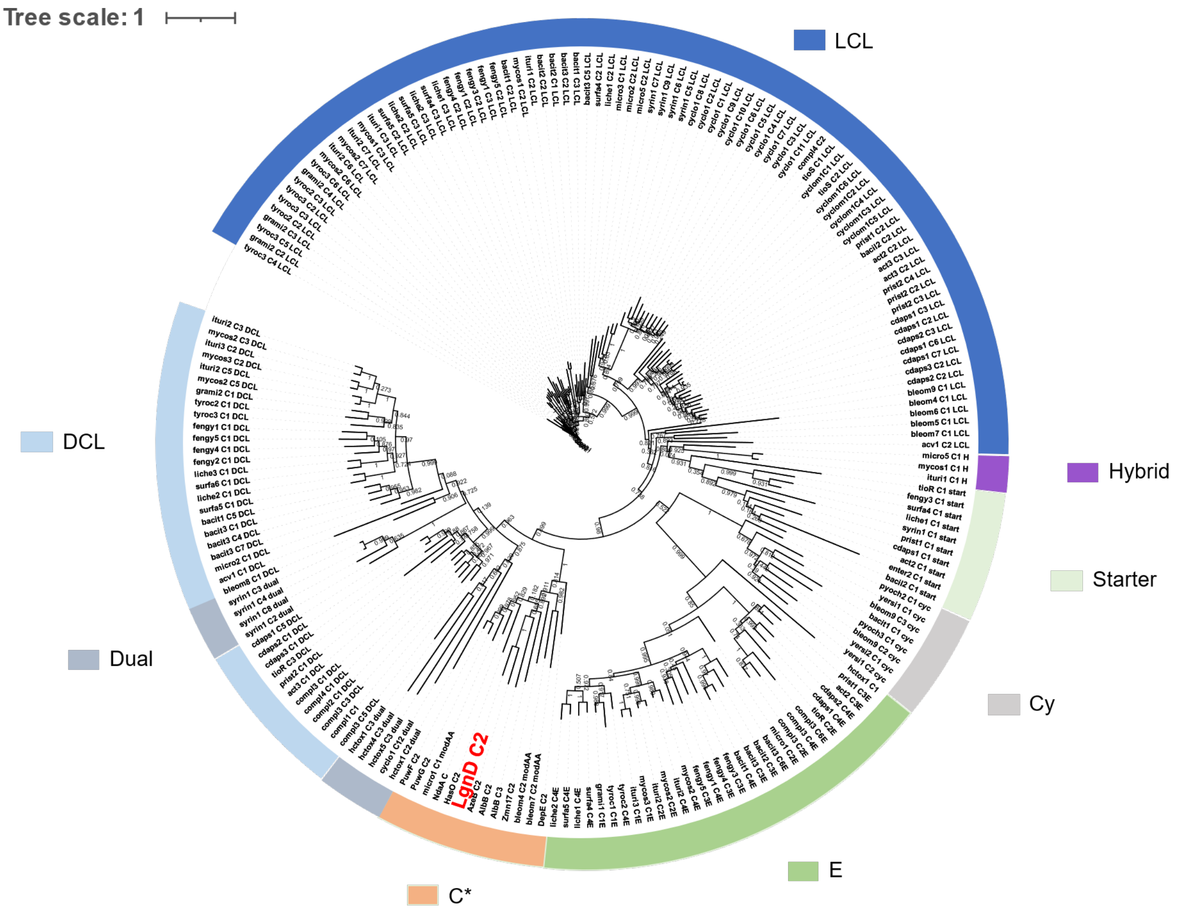

Supplementary Fig. 14. Phylogenetic analysis of C domains available in public domain. The C_2 domain in LgnD belongs to a unique group of C domains (C*) which is represented by the modified AA-type C domains from McyA for microcystin biosynthesis and Bleom4 and Bleom7 for bleomycin biosynthesis in NaPDoS database[2]. Nine C*-type domains from NRPS proteins including AlbB (WP_150244304.1), Zmn17 (CCM44337.1), DepE (ABP57749.1), HasO (CZT62784.1), NdaA (ATP76243.1), PuwF (AIW82283.1), PuwG (AIW82284.1), AmbE (AAG05690.1) and AzeB (AAG06715.1), were added for detailed analysis. The tree was constructed from NaPDoS and was visualized using Interactive Tree Of Life (iTOL) v4[3]. LCL: condensation between two L amino acids; DCL: condensation between a D- and a L-amino acid; Starter: acylation of the first amino acid with a fatty acid, polyketide or other carboxylic acid; Cy: condensation and the subsequent cyclization; E: epimerization; Dual: epimerization and condensation; Hybrid: condensation of amino acid to polyketide. The information of C domains used in this analysis are found in NaPDoS database[2].

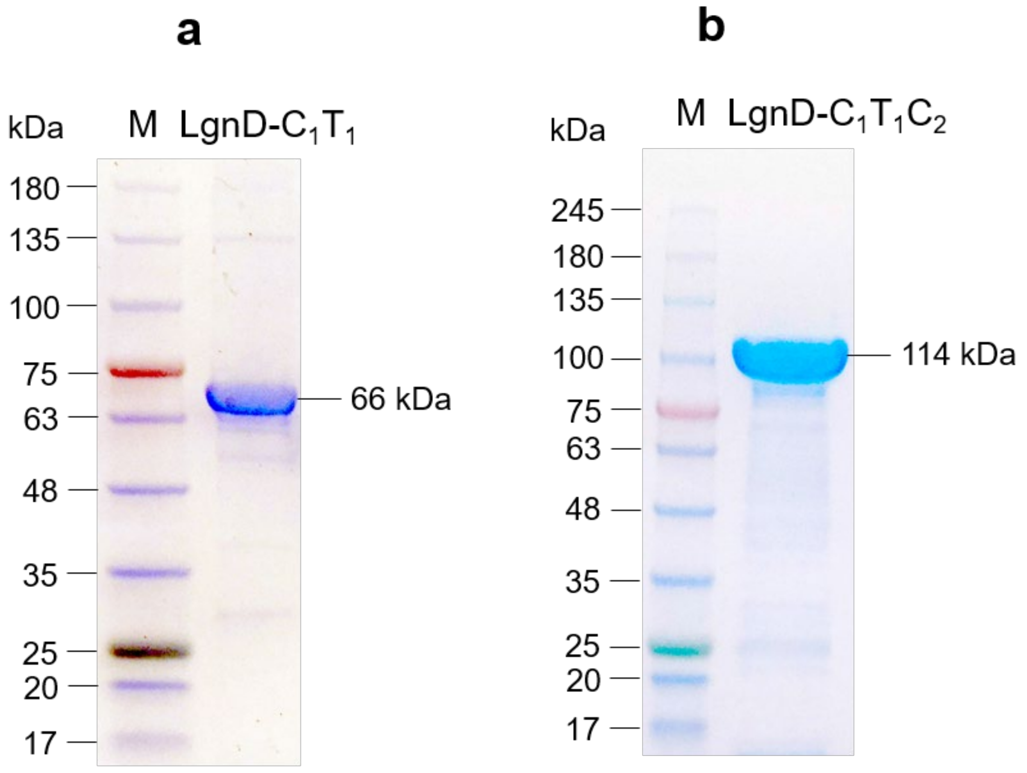

Supplementary Fig. 15. SDS-PAGE analysis of recombinant truncated N-His_6–tagged LgnD C_1-T_1 (**a**) and LgnD C_1-T_1-C_2 (**b**). All experiments were repeated independently with similar results for three times.

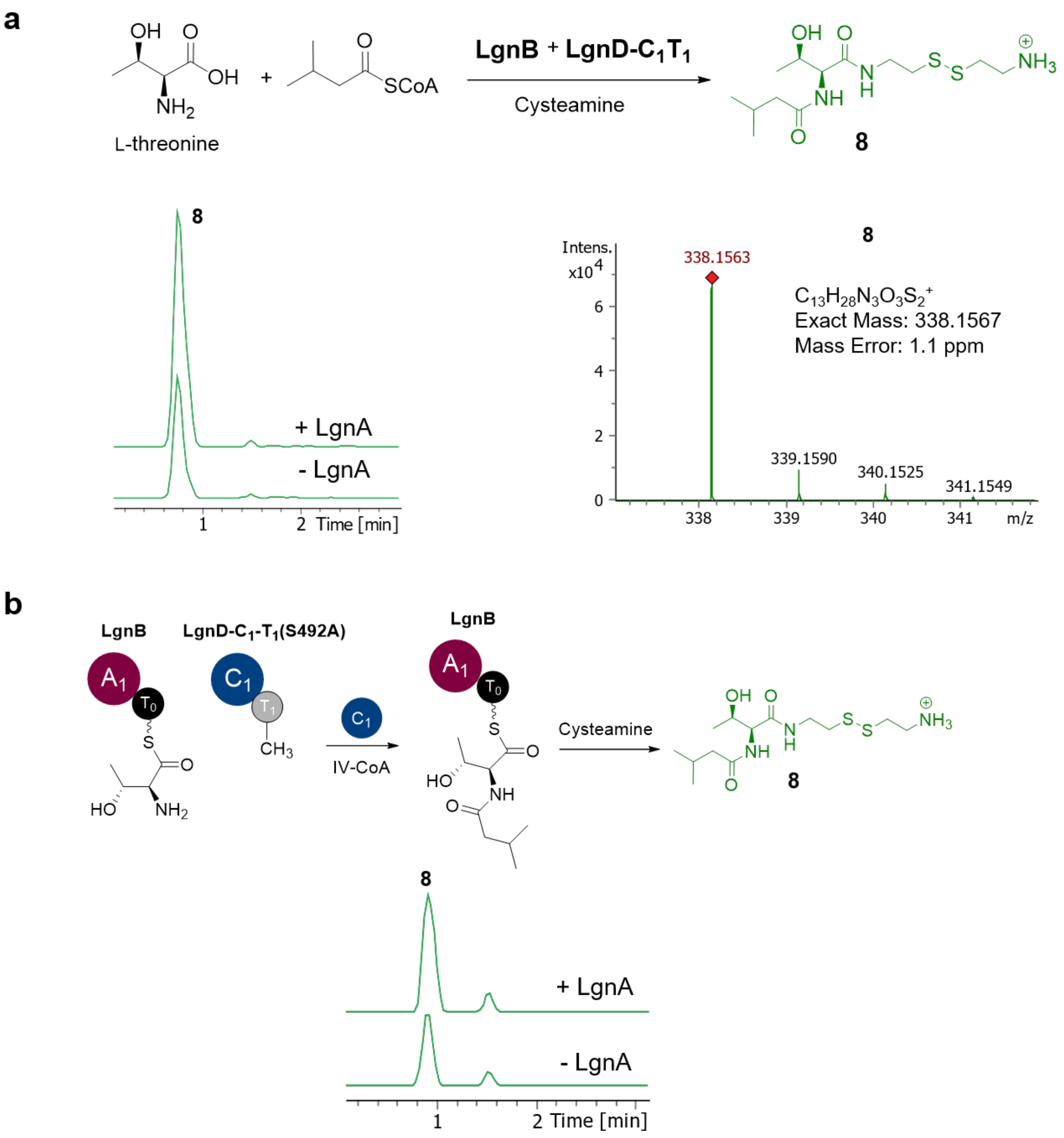

Supplementary Fig. 16. UHPLC-HR-ESI-MS analysis of chemical offloading of expected NRPS-bound intermediates in one-pot reaction of LgnB and LgnD-C_1T_1 or LgnD-C_1T_1(S492A) variant with substrates in the presence or absence of LgnA. **a**. LC-MS trace (left) and MS spectrum (*m/z* 338.1567 $[M+H]^+$) (right) of **8** in an assay of LgnD-C_1T_1 . **b**. LC-MS trace of **8** in an assay of LgnD-C_1T_1(S492A) variant. All LC-MS traces were represented in the same scale of ion intensity (10^4).

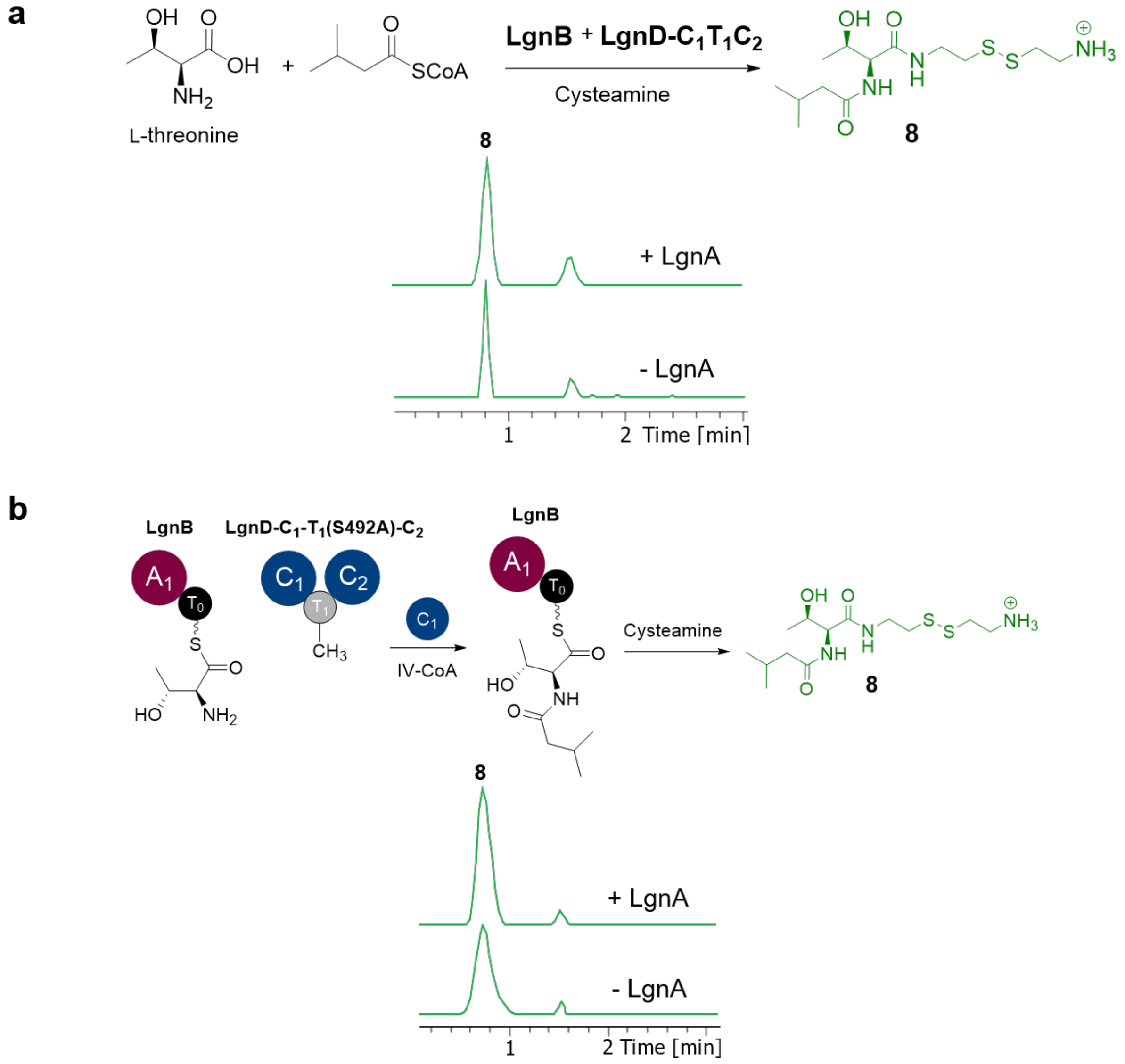

Supplementary Fig. 17. UHPLC-HR-ESI-MS analysis of chemical offloading of expected NRPS-bound intermediates in one-pot reaction of LgnB and LgnD-C_1-T_1-C_2 or LgnD-C_1-T_1S492A-C_2 variant with substrates in the presence or absence of LgnA. **a**. LC-MS trace (*m/z* 338.1567 $[M+H]^+$) of **8** in an assay of LgnD-C_1-T_1-C_2. **b**. LC-MS trace of **8** in an assay of LgnD-C_1-T_1S492A-C_2 variant. All LC-MS traces were represented in the same scale of ion intensity (10^4).

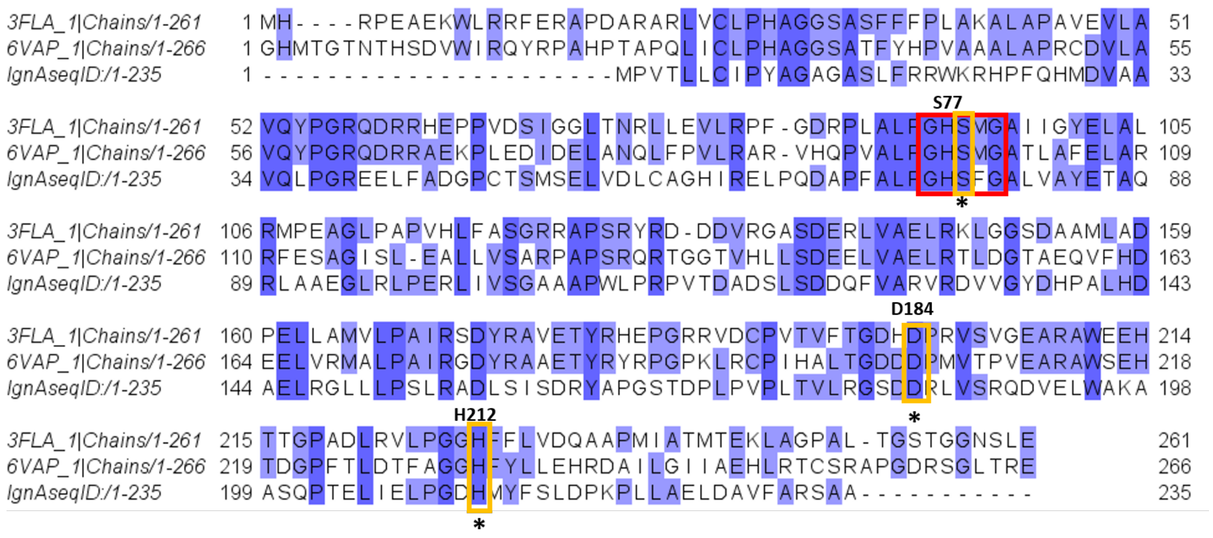

3FLA_1|Chains/1-261 1 MH----RPEAEKWLRRFERAPDARARLVCLPHAGGSASFFFPLAKALAPAVEVLA 51
6VAP_1|Chains/1-266 1 GHMTGTNTHSDVWIRQYRPAHPTAPQLICLPHAGGSATFYHPVAAALAPRCDVLA 55
IgnAseqID:/1-235 1 ----------------------MPVTLLCIPYAGAGASLFRRWKRHPFQHMDVAA 33
S77
3FLA_1|Chains/1-261 52 VQYPGRQDRRHEPPVDSIGGLTNRLLEVLRPF-GDRPLALFGHSMGAIIGYELAL 105
6VAP_1|Chains/1-266 56 VQYPGRQDRRAEKPLEDIDELANQLFPVLRAR-VHQPVALFGHSMGATLAFELAR 109
IgnAseqID:/1-235 34 VQLPGREELFADGPCTSMSELVDLCAGHIRELPQDAPFALFGHSFGALVAYETAQ 88
*
3FLA_1|Chains/1-261 106 RMPEAGLPAPVHLFASGRRAPSRYRD-DDVRGASDERLVAELRKLGGSDAAMLAD 159
6VAP_1|Chains/1-266 110 RFESAGISL-EALLVSARPAPSRQRTGGTVHLLSDEELVAELRTLDGTAEQVFHD 163
IgnAseqID:/1-235 89 RLAAEGLRLPERLIVSGAAAPWLPRPVTDADSLSDDQFVARVRDVVGYDHPALHD 143
D184
3FLA_1|Chains/1-261 160 PELLAMVLPAIRSDYRAVETYRHEPGRRVDCPVTVFTGDHDPRVSVGEARAWEEH 214
6VAP_1|Chains/1-266 164 EELVRMALPAIRGDYRAAETYRYRPGPKLRCPIHALTGDDDPMVTPVEARAWSEH 218
IgnAseqID:/1-235 144 AELRGLLLPSLRADLSISDRYAPGSTDPLPVPLTVLRGSDDRLVSRQDVELWAKA 198
*
H212
3FLA_1|Chains/1-261 215 TTGPADLRVLPGGHFFLVDQAAPMIATMTEKLAGPAL-TGSTGGNSLE 261
6VAP_1|Chains/1-266 219 TDGPFTLDTFAGGHFYLLEHRDAILGIIAEHLRTCSRAPGDRSGLTRE 266
IgnAseqID:/1-235 199 ASQPTELIELPGDHMYFSLDPKPLLAELDAVFARSAA----------- 235
*

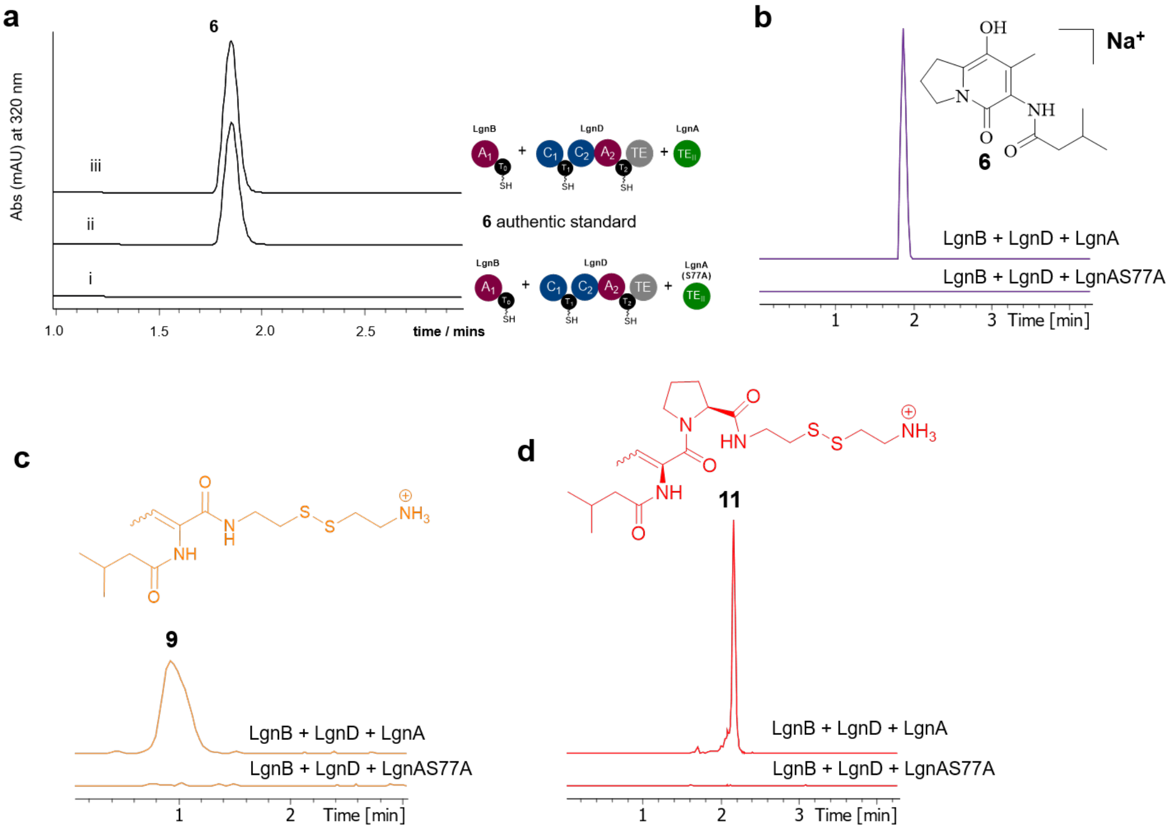

Supplementary Fig. 19. HPLC and HR-MS analyses of the production of **6** and key intermediate adducts, **9** and **11**. in assays of LgnB and LgnD with substrates in the presence of LgnA and the LgnA(S77A) variant. **a**. HPLC traces of (i) the reaction mixtures from an assay of *holo*-LgnB, *holo*-LgnD together with ATP, Thr, Pro, and IV-CoA in the presence of the LgnA(S77A) variant; (ii). The authentic **6**; (iii). the control experiment of the reaction mixtures from an assay of *holo*-LgnB, *holo*-LgnD in the presence of the LgnA. **b**. Extracted ion chromatograms (EICs) of the presence of **6** (m/z 287.1366 $[M+Na]^+$) when LgnA was added (top), and the absence of **6** (bottom) when Lgn(S77A) was added. **c**. HR-MS analyses of expected NRPS-bound dehydrated intermediate adduct, **9**. Extracted ion chromatograms (EICs) of the cystamine-adducted IV-Dhb **9** (*m/z* 320.1461 $[M+H]^+$) in the assays of LgnB and LgnD when LgnA (top) or the LgnA(S77A) variant (bottom) was added, respectively. **d**. HR-MS analyses of expected NRPS-bound dehydrated intermediate adduct, **11**. Extracted ion chromatograms (EICs) of the cystamine-adducted IV-Dhb-Pro **11** (*m/z* 417.1989 $[M+H]^+$) in the assays of LgnB and LgnD when LgnA (top) or the LgnAS77A variant (bottom) was added, respectively.

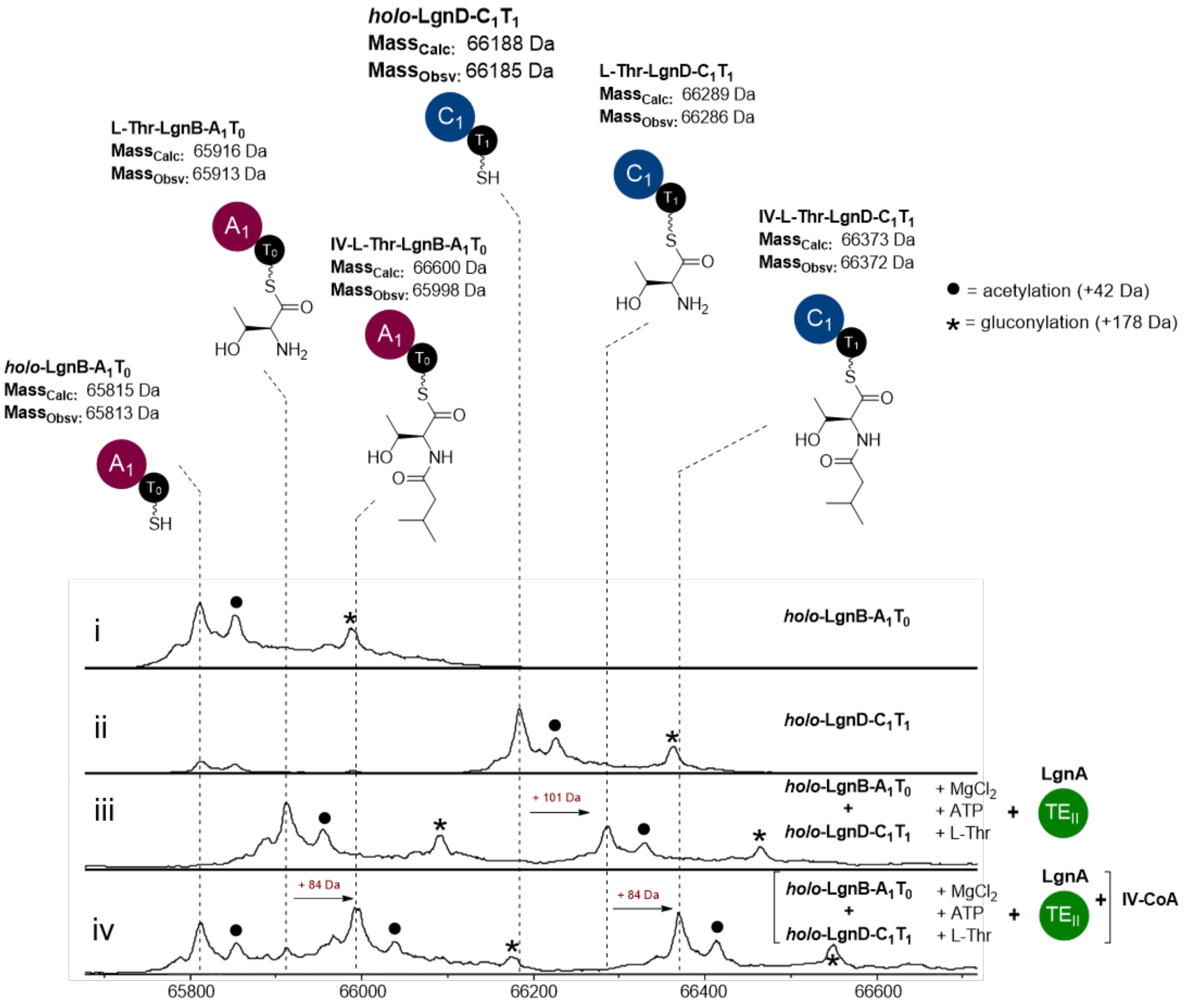

Supplementary Fig. 20. Snapshots of early-stage biosynthetic intermediates, indicating that the formation of IV-Thr unit occur on both T_0 and T_1 domains. Deconvoluted intact protein mass spectra of (**i**)- (**ii**): control spectra of LgnD -C_1-T_1 didomain and LgnB, respectively; (**iii**) a control spectrum of an assay of LgnA, LgnB and LgnD -C_1-T_1 didomain, together with Thr, ATP, Mg^{2+}. (**iv**) an assay of pre-incubation of LgnA, LgnB and LgnD-C_1-T_1 didomain, followed by addition of IV-CoA; Mass shifts corresponding to biosynthetic steps are highlighted with arrows, and proposed intermediates are displayed. Peaks labelled with dots and asterisks indicate N-terminal acetylation and gluconoylation, known post-translational modifications of recombinant heterologous proteins in *E. coli*. Exact measured and observed masses are detailed in **Supplementary Table 3**.

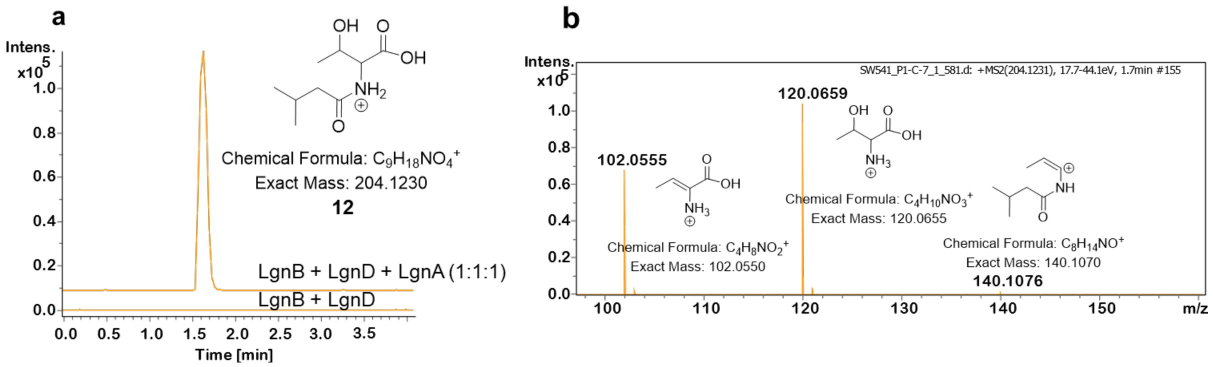

Supplementary Fig. 21. UHPLC-HR-ESI-MS analysis indicated that LgnA specifically removes the aberrant IV-Thr intermediates. **a**. Extracted ion chromatograms (EICs) of the presence of **12** (*m/z* 204.1230 $[M+H]^+$) when LgnA was added in the assay (top) and the absence of **12** when LgnA was omitted. **b**. MS^2 fragmentation data for **12**.

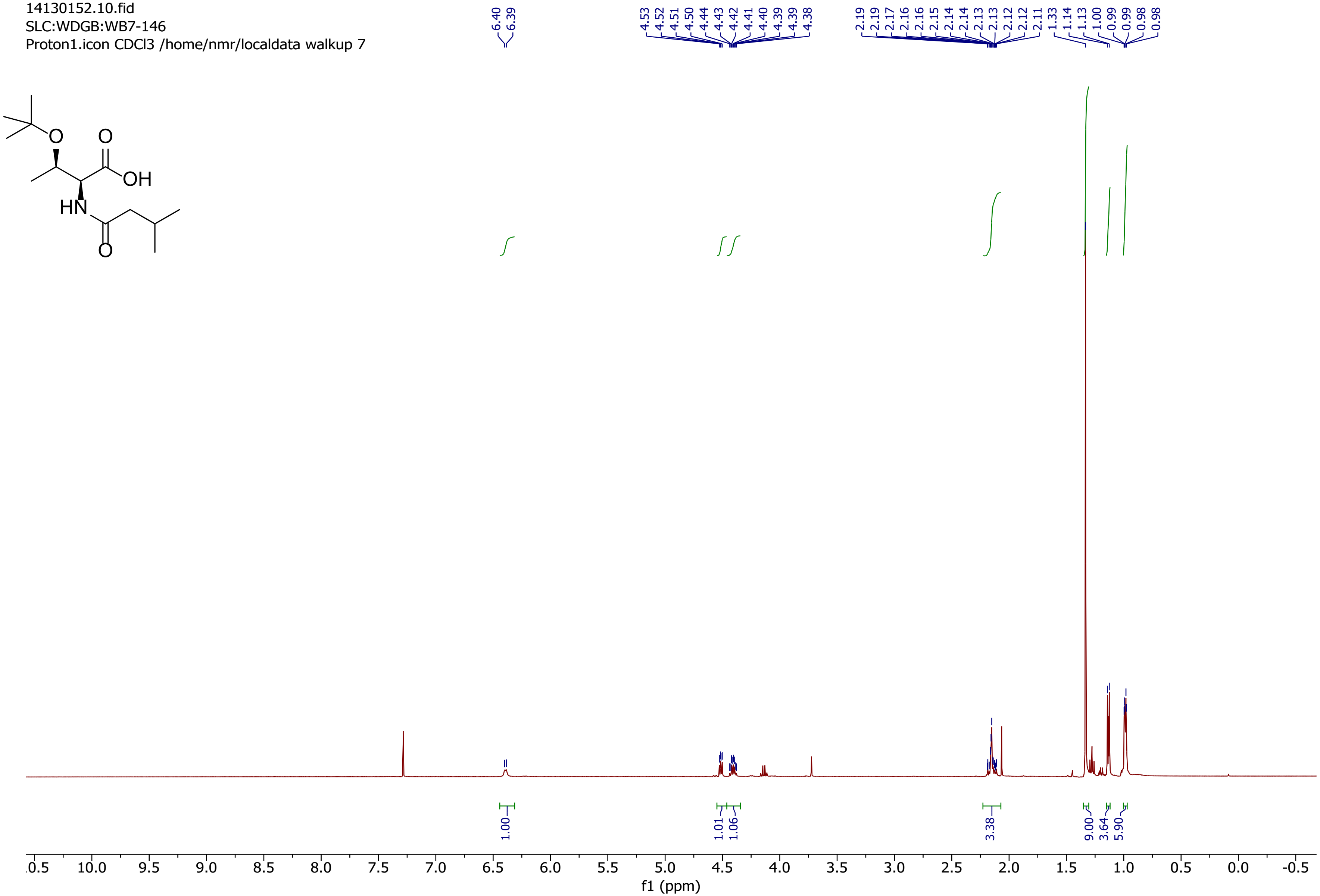

Supplementary Fig 22 a. ^{1}H NMR spectrum of O-*t*Bu-isovaleryl-L-Thr **12a** in $CDCl_3$ (400 MHz, 28°C)

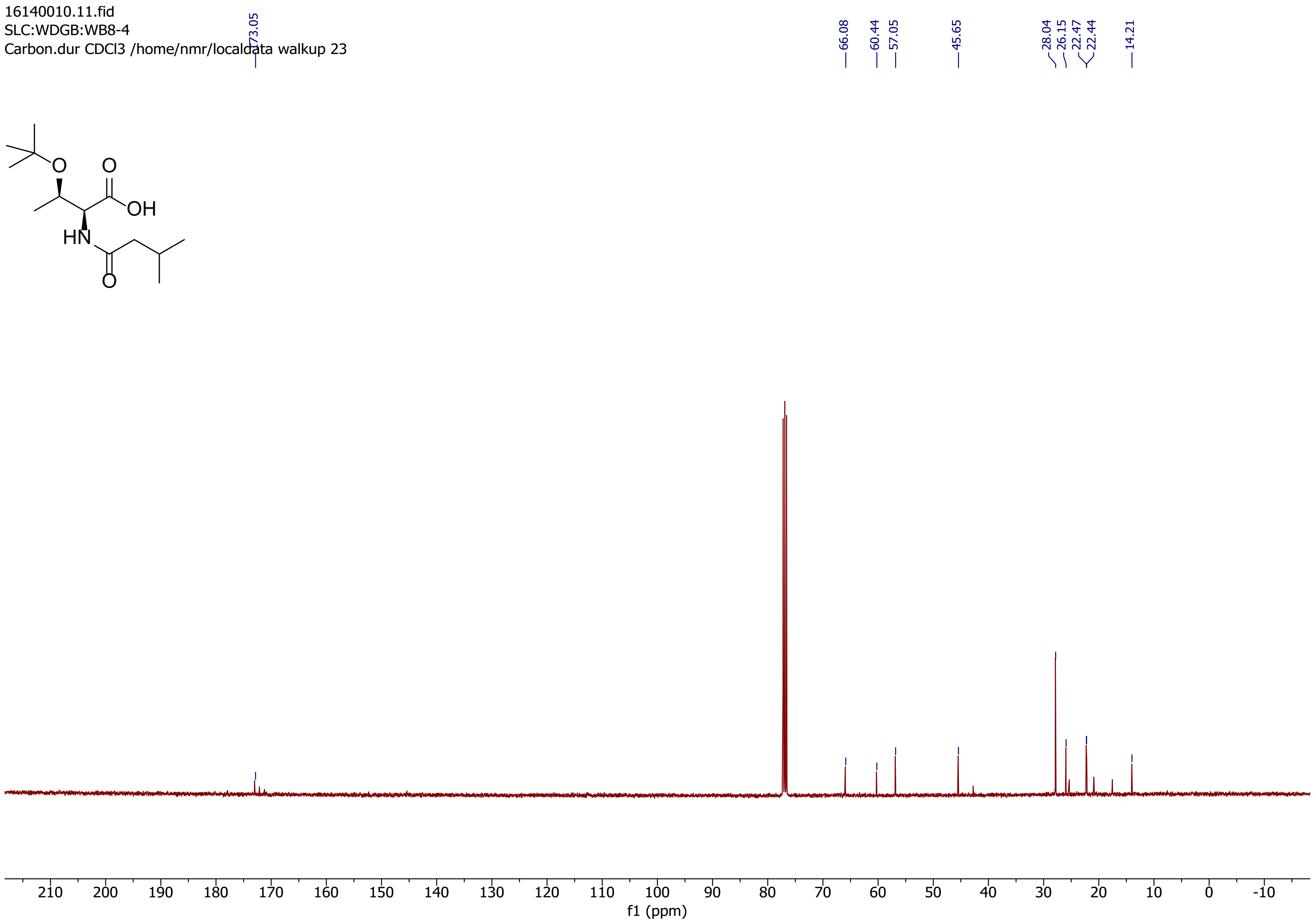

Supplementary Fig 22 b. [12]C NMR spectrum of isovaleryl-O-*t*Bu-L-Thr-SNAC **12a** in $CDCl_3$ (150 MHz, 28°C)

21114433.10.fid
SLC:WDGB:WB8-39 F24-30
Proton.dur CDCl3 /home/nmr/localdata walkup 5

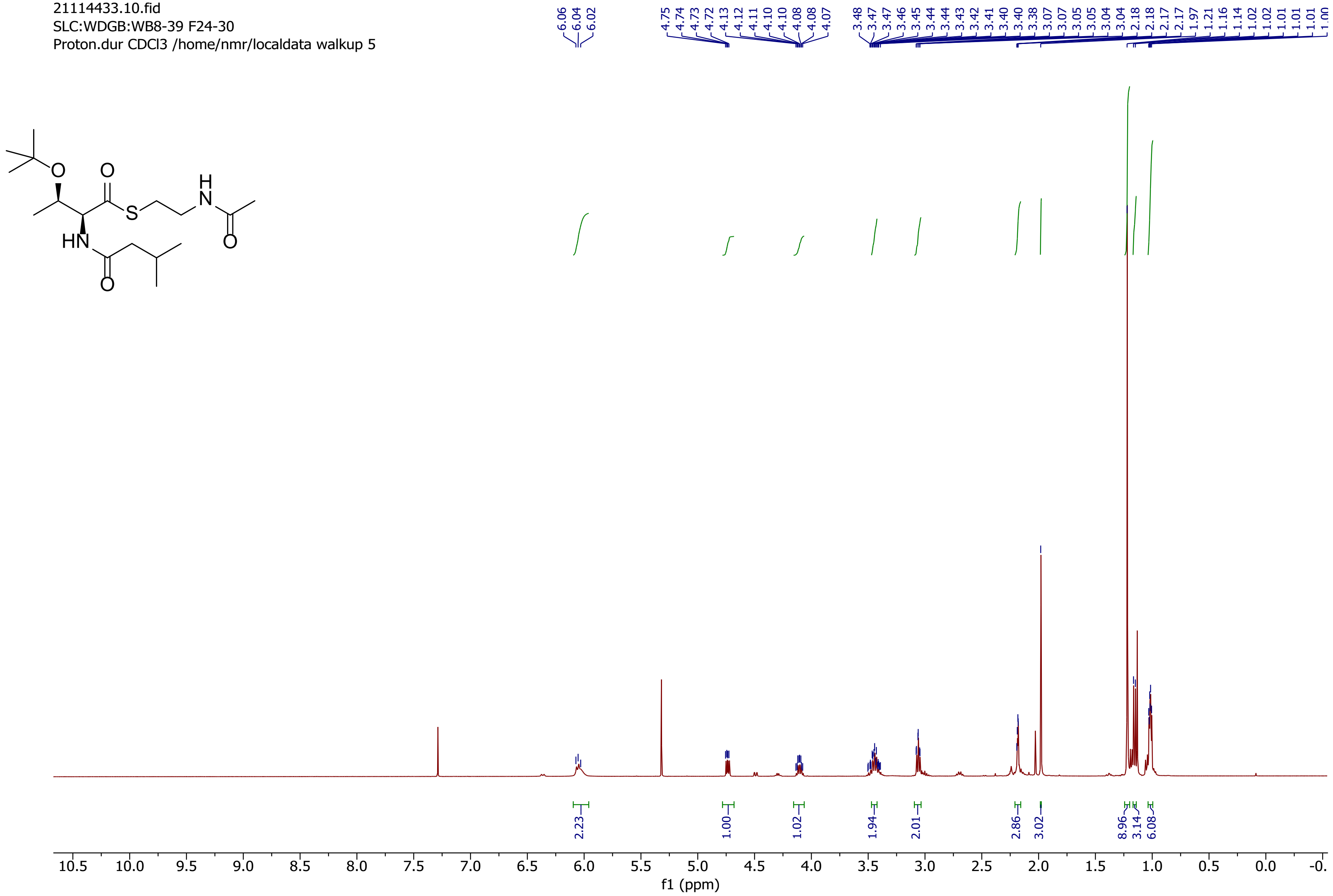

Supplementary Fig 23 a. ^{1}H NMR spectrum of isovaleryl-O-*t*Bu-L-Thr-SNAC **12b** in $CDCl_3$ (400 MHz, 28°C)

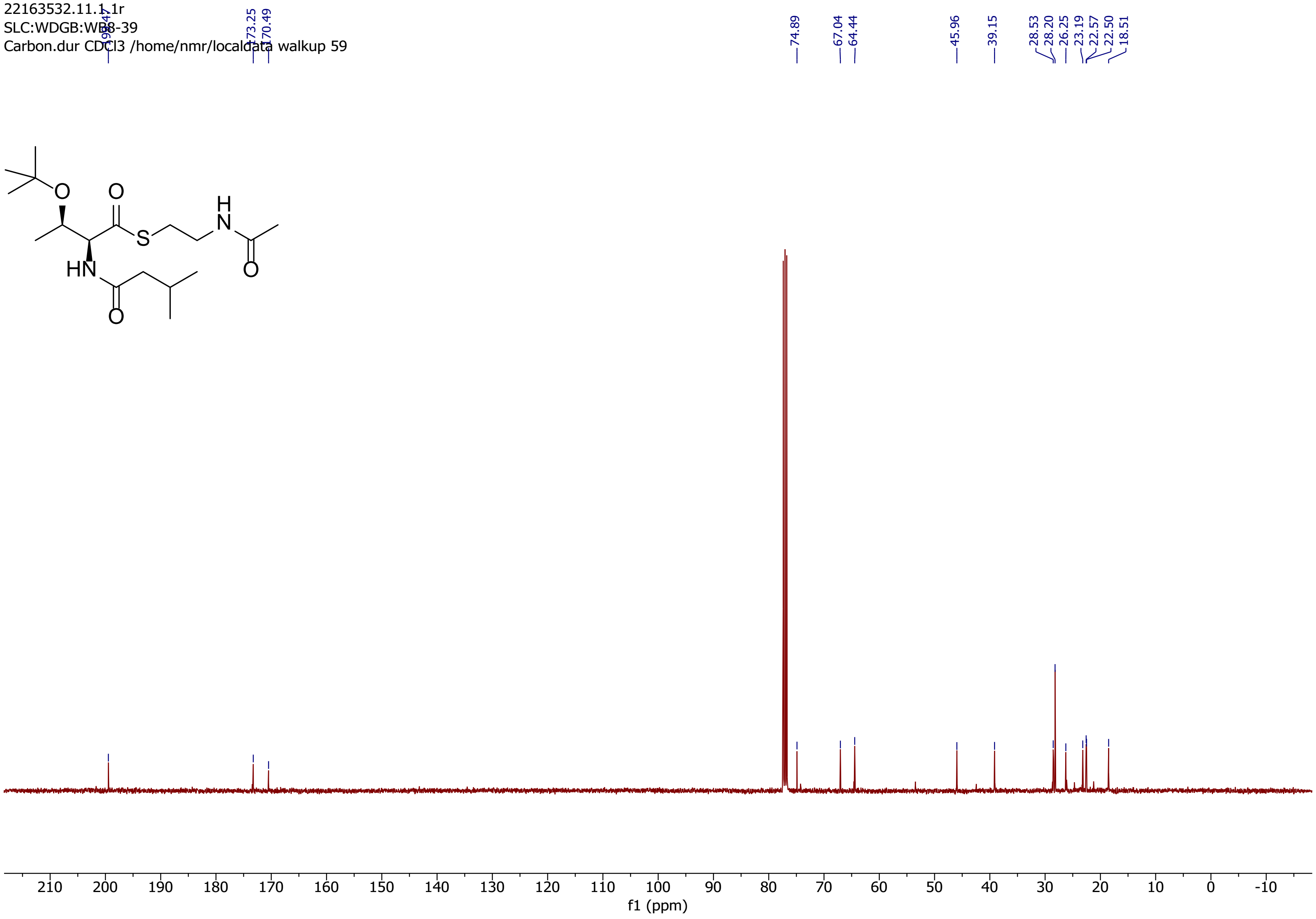

Supplementary Fig 23 b. ^{12}C NMR spectrum of isovaleryl-O-*t*Bu-L-Thr-SNAC **12b** in $CDCl_3$ (150 MHz, 28°C)

Elemental Composition Report **Page 1**

Single Mass Analysis
Tolerance = 3.0 mDa / DBE: min = -1.5, max = 50.0
Element prediction: Off
Number of isotope peaks used for i-FIT = 3

Monoisotopic Mass, Even Electron Ions
427 formula(e) evaluated with 3 results within limits (up to 50 closest results for each mass)
Elements Used:
C: 0-50 H: 0-50 N: 0-4 O: 0-6 S: 0-2
William Brittain
WB8-39 F24-30 379 (3.191) Cm (371:383)

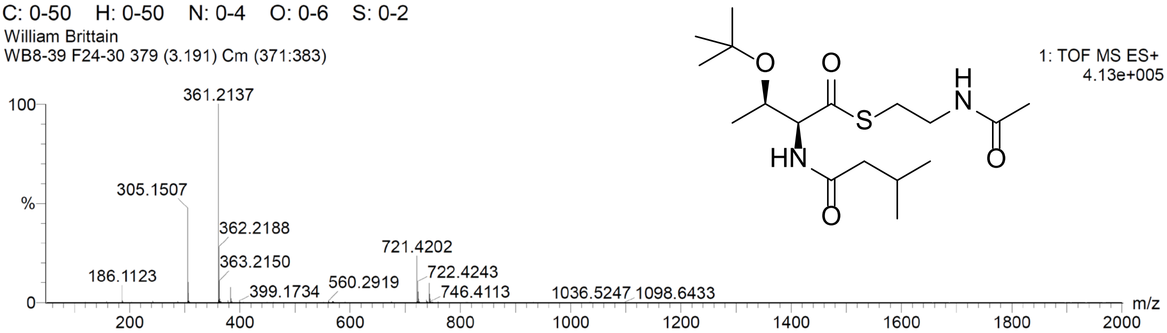

Minimum:				-1.5			
Maximum:		3.0	5.0	50.0			

Mass	Calc. Mass	mDa	PPM	DBE	i-FIT	i-FIT (Norm)	Formula
361.2137	361.2127	1.0	2.8	7.5	755.4	6.1	C20 H29 N2 O4
	361.2121	1.6	4.4	-1.5	752.2	3.0	C12 H33 N4 O6 S
	361.2161	-2.4	-6.6	2.5	749.3	0.1	C17 H33 N2 O4 S

Supplementary Fig 23 c. Elemental analysis of isovaleryl-O-*t*Bu- L-Thr -SNAC **12b**.

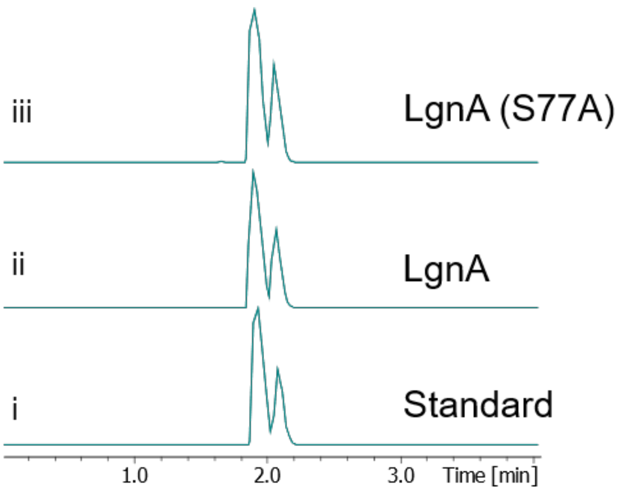

Supplementary Fig. 24 LS-MS analysis of (i) synthetic IV-Thr-SNAC **14a**, (ii) the biochemical assay of LgnA with **14a**, and (iii) the biochemical assay of LgnA(S77A) variant with **14a**. All LC-MS traces were represented in the same scale of ion intensity (10^6).

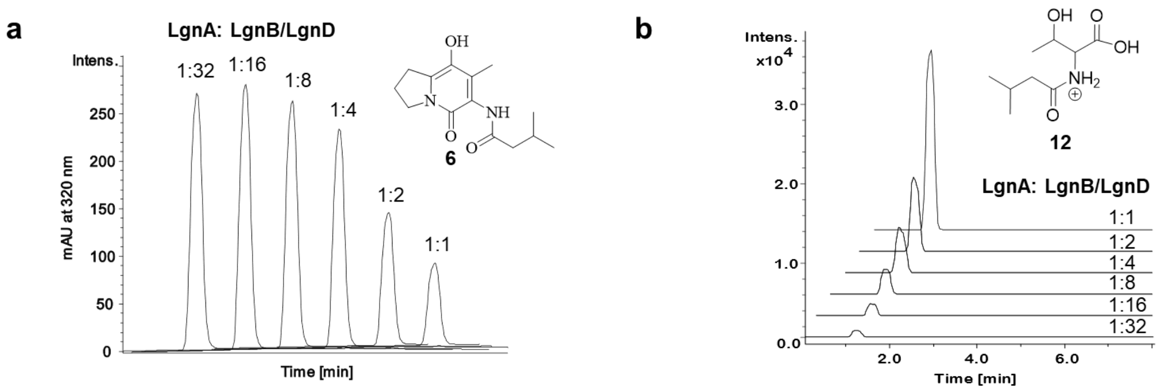

Supplementary Fig. 25. UHPLC-HR-ESI-MS analysis indicated that the ratio of LgnA and LgnB/LgnD is important to the production of **6** and the hydrolytic reaction to generate a shunt product **12**. **a**. Increasing the concentrations of LgnA in the assay led to the increased production of the hydrolytic product, IV-Thr-COOH **12**. (**b**) Increasing the concentrations of LgnA in the assay led to the decreased production of the NRPS final product, **6**.

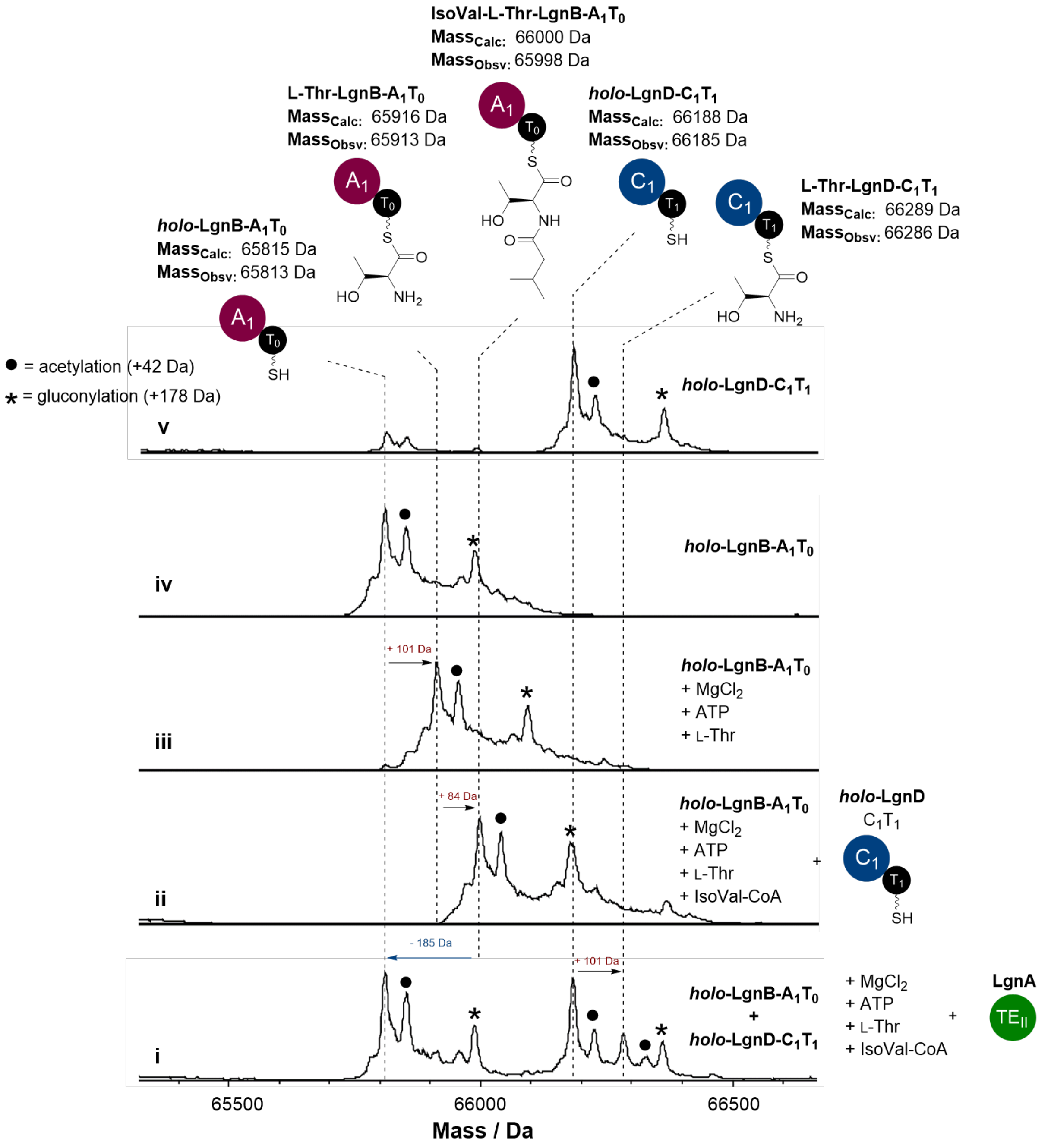

Supplementary Fig. 26. Snapshots of early-stage biosynthetic intermediates in an assay of a high molecular ratio of LgnA with LgnB and LgnD-C_1-T_1, indicating that the high concentration of LgnA reduces the efficiency of the formation of IV-Thr unit. Deconvoluted intact protein mass spectra of (**i**) an assay of LgnB and LgnD-C_1-T_1 together with addition of excess LgnA (the molecular ratio of LgnA to LgnB/LgnD-C_1-T_1 was 1:4); (**ii**) an control assay of LgnB and LgnD-C_1-T_1 together with substrates in the absence of LgnA; (**iii**) a control assay of LgnB together with substrates; (**iv**)-(**v**) controls of *holo*-LgnB and *holo*-LgnD-C_1-T_1. Mass shifts corresponding to biosynthetic steps are highlighted with arrows, and proposed intermediates are displayed. Peaks labelled with dots and asterisks indicate N-terminal acetylation and gluconoylation, known post-translational modifications of recombinant heterologous proteins in *E. coli*. Exact measured and observed masses are detailed in **Supplementary Table 3**.

a

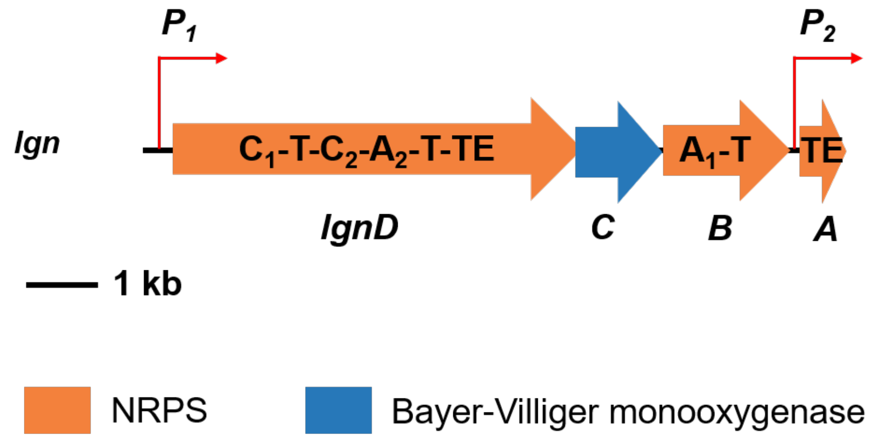

b

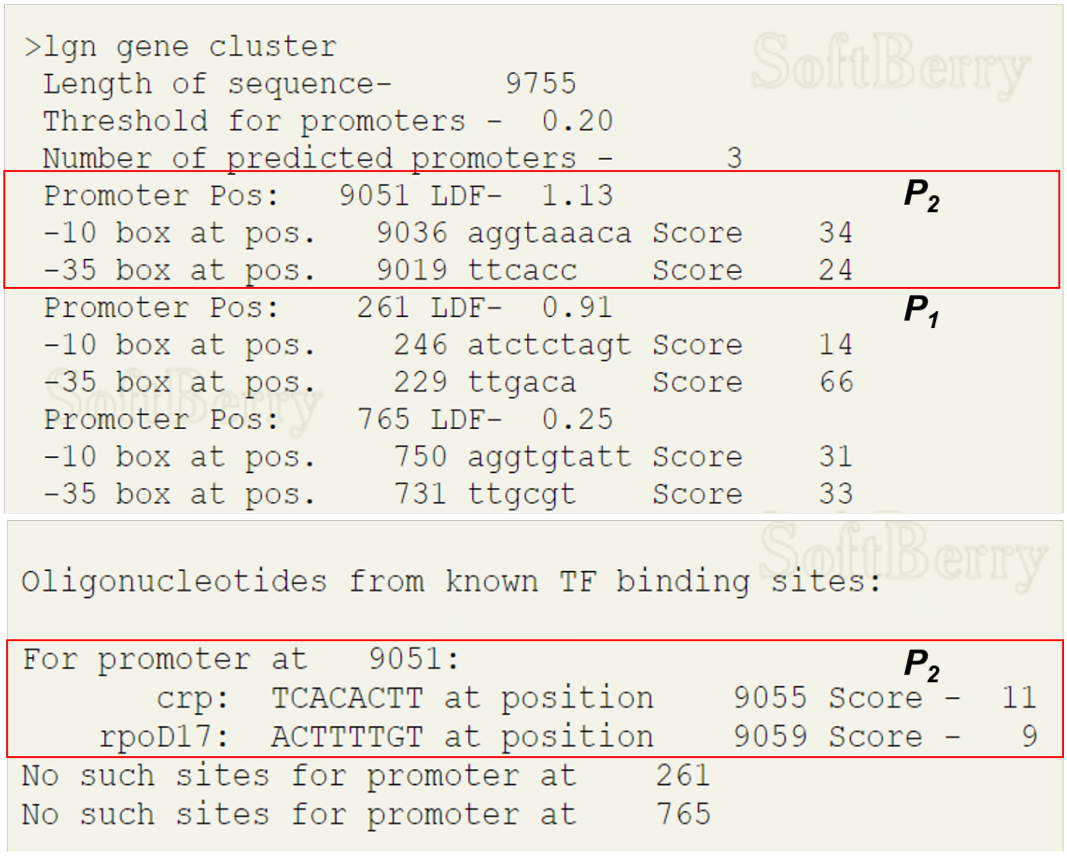

```
>lgn gene cluster
 Length of sequence-      9755
 Threshold for promoters -  0.20
 Number of predicted promoters -       3
 Promoter Pos:   9051 LDF-  1.13                P2
 -10 box at pos.   9036 aggtaaaca Score   34
 -35 box at pos.   9019 ttcacc    Score   24
 Promoter Pos:    261 LDF-  0.91                P1
 -10 box at pos.    246 atctctagt Score   14
 -35 box at pos.    229 ttgaca    Score   66
 Promoter Pos:    765 LDF-  0.25
 -10 box at pos.    750 aggtgtatt Score   31
 -35 box at pos.    731 ttgcgt    Score   33

Oligonucleotides from known TF binding sites:

For promoter at   9051:                          P2
       crp:  TCACACTT at position    9055 Score -  11
    rpoD17:  ACTTTTGT at position    9059 Score -   9
No such sites for promoter at    261
No such sites for promoter at    765
```

Supplementary Fig. 27. Bioinformatic analysis of the minimal BGC that directs the production of legonmycin A, **5**, suggested that there are two putative promoter regions, one for *lgnB-D* (P_1), and the other for *lgnA* gene (P_2). **a.** solid red arrows represent the promoter regions of *lgn* predicted by BPROM from Softberry. [6] **b.** The BPROM output shows the positions of predicted promoters, their scores and the elements of Transcriptional factor binding sites for P_2.

a

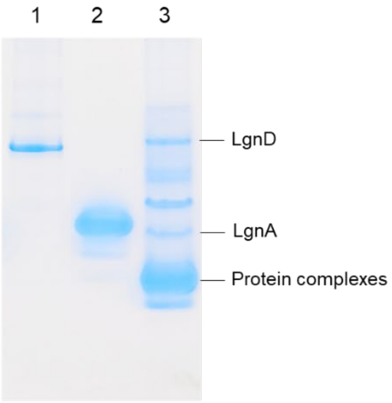

b

1. 1::bms|BMS050011|Xuan LgnD-CHis [] Mass: 207559 Score: 606 Matches: 15(15) Sequences: 14(14)
LgnD-CHis []
☐ Check to include this hit in error tolerant search or archive report

	Query	Observed	Mr(expt)	Mr(calc)	ppm	Miss	Score	Expect	Rank	Unique	Peptide
☑	8	440.7434	879.4723	879.4702	2.42	0	44	9.5e-05	1	U	R.TSFVIEGK.V
☑	11	448.7833	895.5520	895.5491	3.24	0	46	2.5e-05	1	U	R.LPLADVLR.D
☑	12	455.7446	909.4747	909.4708	4.25	0	33	0.0012	1	U	K.FEFIAQR.G
☑	14	481.7892	961.5637	961.5596	4.26	0	55	4.9e-06	1	U	R.LYDAVLLR.A
☑	16	494.2490	986.4835	986.4781	5.50	0	36	0.0004	1	U	R.QLQQEGER.T
☑	18	499.2779	996.5411	996.5352	5.97	0	41	0.00018	1	U	R.AQPEAAAAIR.S
☑	19	505.7332	1009.4518	1009.4465	5.27	0	48	2.4e-05	1	U	R.ASESFAQDR.Q
☑	21	519.2618	1036.5090	1036.5012	7.57	0	(54)	1.5e-05	1	U	R.YGVDIPMSR.F
☑	23	527.2582	1052.5019	1052.4961	5.54	0	58	4.9e-06	1	U	R.YGVDIPMSR.F + Oxidation (M)
☑	27	612.8332	1223.6518	1223.6397	9.90	0	60	1.9e-06	1	U	R.SVQELASSYLK.A
☑	29	659.8362	1317.6579	1317.6412	12.7	0	73	1.2e-07	1	U	R.DELLALESADSR.E
☑	35	676.8551	1351.6957	1351.6772	13.7	0	47	6.5e-05	1	U	R.LSAFEDAFPLSR.A
☑	38	761.4011	1520.7875	1520.7624	16.6	0	85	8.6e-09	1	U	R.SGGASATFDPLVPFR.T
☑	40	811.4808	1620.9471	1620.9199	16.8	0	52	6.2e-06	1	U	R.GVELGVLPPGSSAAIVR.R
☑	42	580.9674	1739.8803	1739.8625	10.3	0	94	8.9e-10	1	U	R.AHQADEVTLGVAMLNR.H + Oxidation (M)

2. 1::bms|BMS050009|Xuan His-LgnA [] Mass: 29570 Score: 356 Matches: 7(7) Sequences: 7(7)
His-LgnA []
☐ Check to include this hit in error tolerant search or archive report

	Query	Observed	Mr(expt)	Mr(calc)	ppm	Miss	Score	Expect	Rank	Unique	Peptide
☑	4	434.7860	867.5575	867.5542	3.87	0	36	0.00024	1	U	R.GLLLPSLR.A
☑	7	438.7258	875.4371	875.4348	2.56	0	80	3.8e-08	1	U	R.ADLSISDR.Y
☑	17	494.7616	987.5087	987.5025	6.25	0	55	1.3e-05	1	U	R.QDVELWAK.A
☑	44	898.5190	1795.0233	1794.9880	19.7	0	72	6.7e-08	1	U	R.YAPGSTDPLPVPLTVLR.G
☑	45	602.9703	1805.8892	1805.8697	10.8	0	50	2.4e-05	1	U	R.DVVGYDHPALHDAELR.G
☑	46	606.9801	1817.9183	1817.8995	10.3	0	81	1.7e-08	1	U	R.HPFQHMDVAAVQLPGR.E + Oxidation (M)
☑	47	516.2703	2061.0519	2061.0392	6.19	1	77	4.4e-08	1	U	R.VRDVVGYDHPALHDAELR.G

Supplementary Fig. 28. Native PAGE analysis of protein-protein complex between LgnA and LgnD. **a**. Lane 1. LgnD, Lane 2, LgnA; Lane 3. The mixture of LgnA and LgnD (1:1). **b**. MS-MS sequencing confirmed that the protein complexes contain LgnD and LgnA mixture. All experiments were repeated independently with similar results for three times.

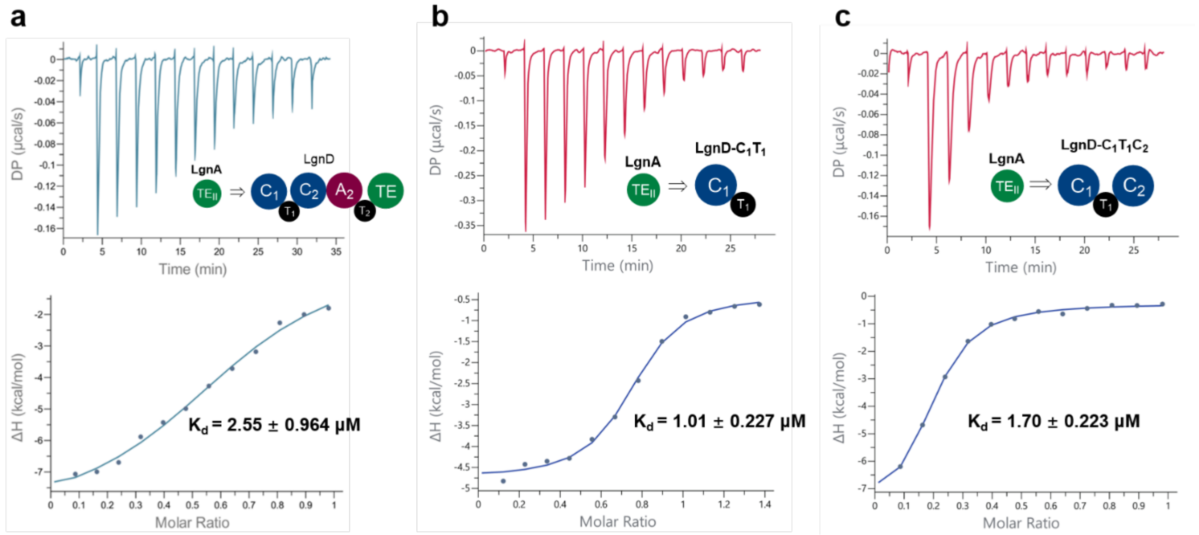

Supplementary Fig. 29. ITC analyses of LgnA titrated with LgnD or truncated forms of LgnD, showing direct interaction with the megasynthetase. In each case, *top* shows raw thermograms, and *bottom* shows the integrated binding enthalpies as isotherm plots for LgnA titrated with LgnD (**a**), LgnA titrated with LgnD C_1-T_1 didomain (**b**), LgnA titrated with LgnD C_1-T_1-C_2 tridomain (**c**).

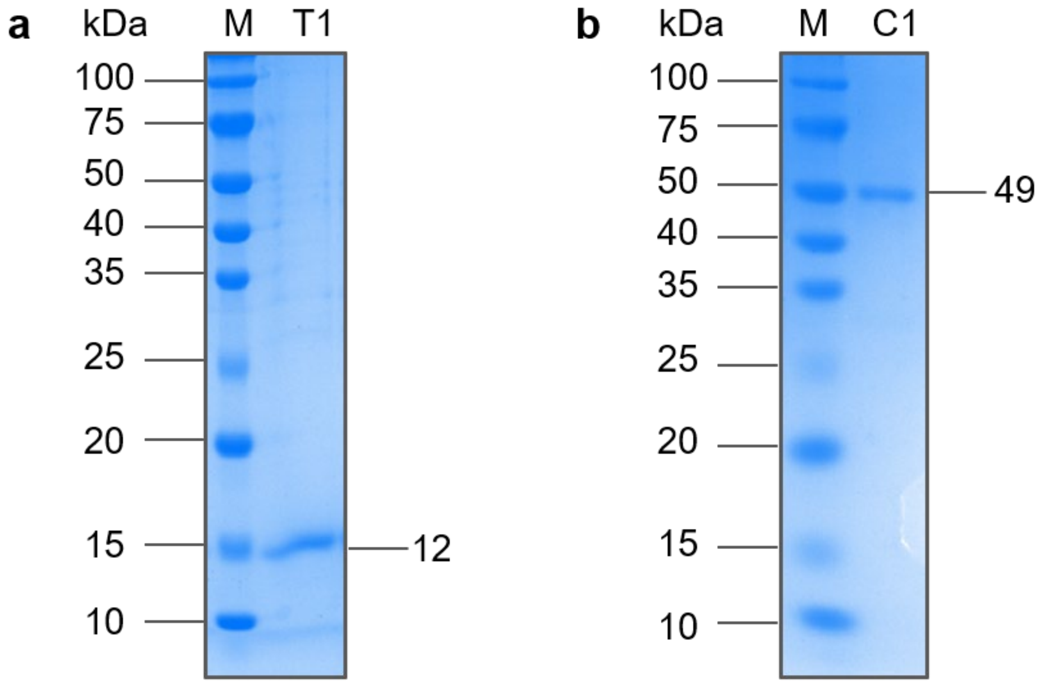

Supplementary Fig. 30. SDS page analysis of LgnD-T_1 (**a**) and LgnD-C_1 (**b**). M: protein ladder. All experiments were repeated independently with similar results for three times.

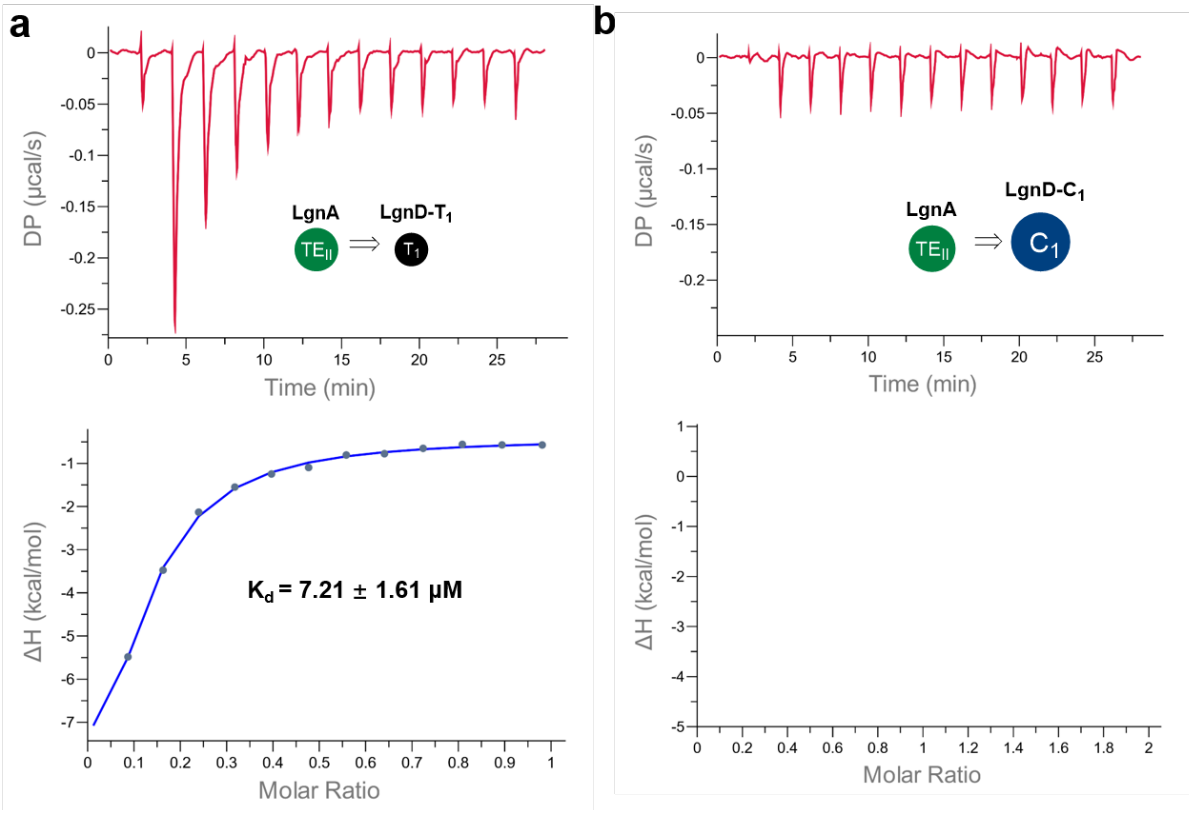

Supplementary Fig. 31. ITC measurements of LgnA titrated with LgnD-T_1 (**a**), and LgnD-C_1 (**b**). In each case, *top* shows raw thermograms, and *bottom* shows the integrated binding enthalpies as isotherm plots.

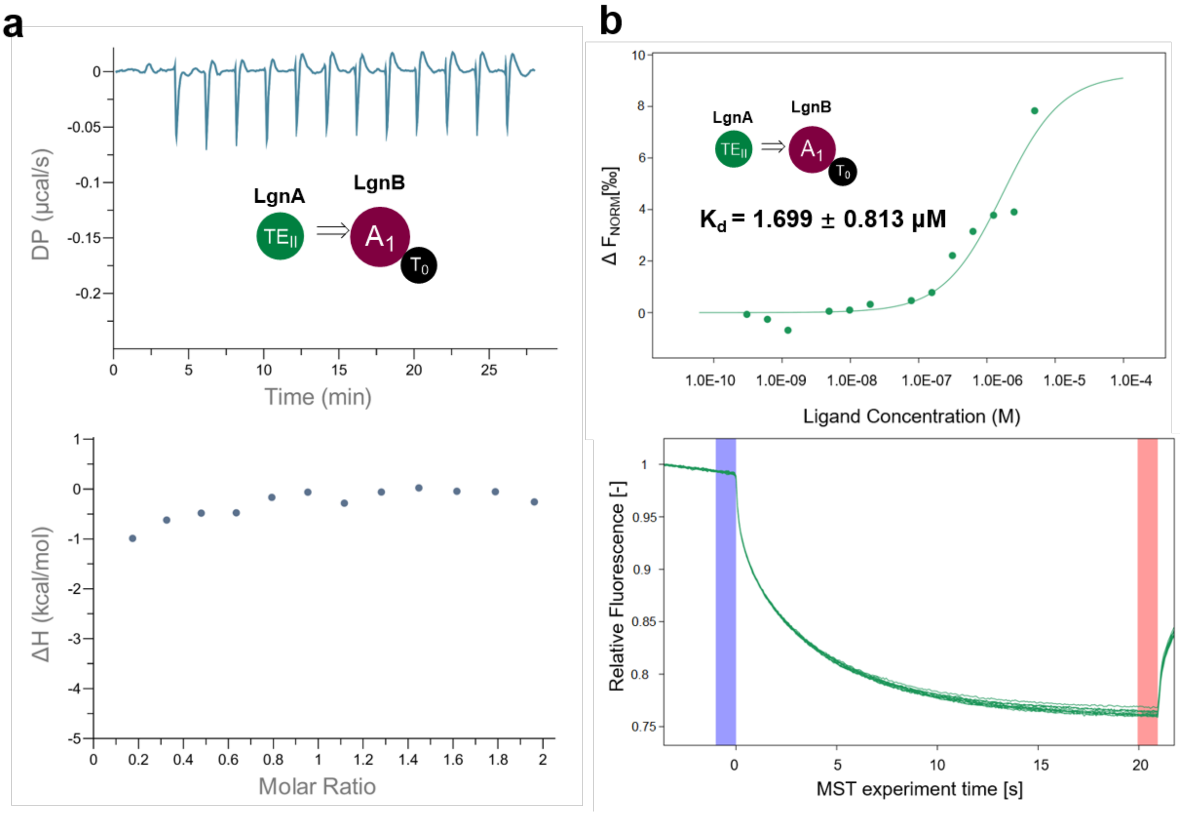

Supplementary Fig. 32. ITC and microscale thermophoresis (MST) assays of LgnA titrated with LgnB didomain. (**a**). ITC measurement of LgnA titrated with *apo*-LgnB. *top* shows raw thermograms, and *bottom* shows the integrated binding enthalpies as isotherm plots. (**b**). MST measurement of LgnA titrated with *apo*-LgnB. *Top*. The binding curve for measurement of specific interaction were derived from the change in the thermophoretic signal upon LgnA titration to a constant concentration (25 nM) of fluorescently labelled LgnB. The curves were fitted to K_d model and yielded K_d = 1.669 ± 0.813 µM. *Bottom*. The changes of relative fluorescence intensity were displayed over time with cold region (0 s) and hot region (20 s) to determine the K_d of the interaction.

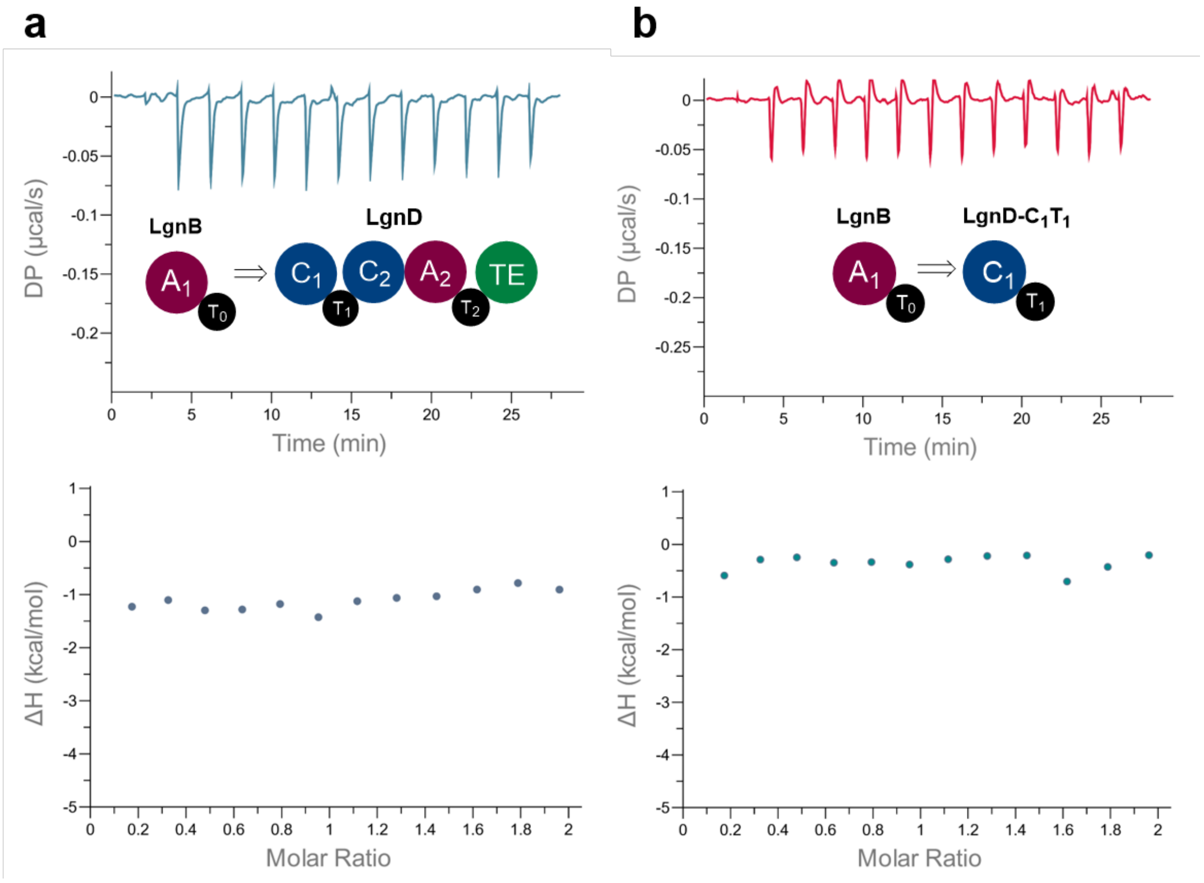

Supplementary Fig. 33. ITC measurements of LgnB titrated with LgnD (**a**), and LgnD-C_1-T_1 (**b**). In each case, *top* shows raw thermograms, and *bottom* shows the integrated binding enthalpies as isotherm plots.

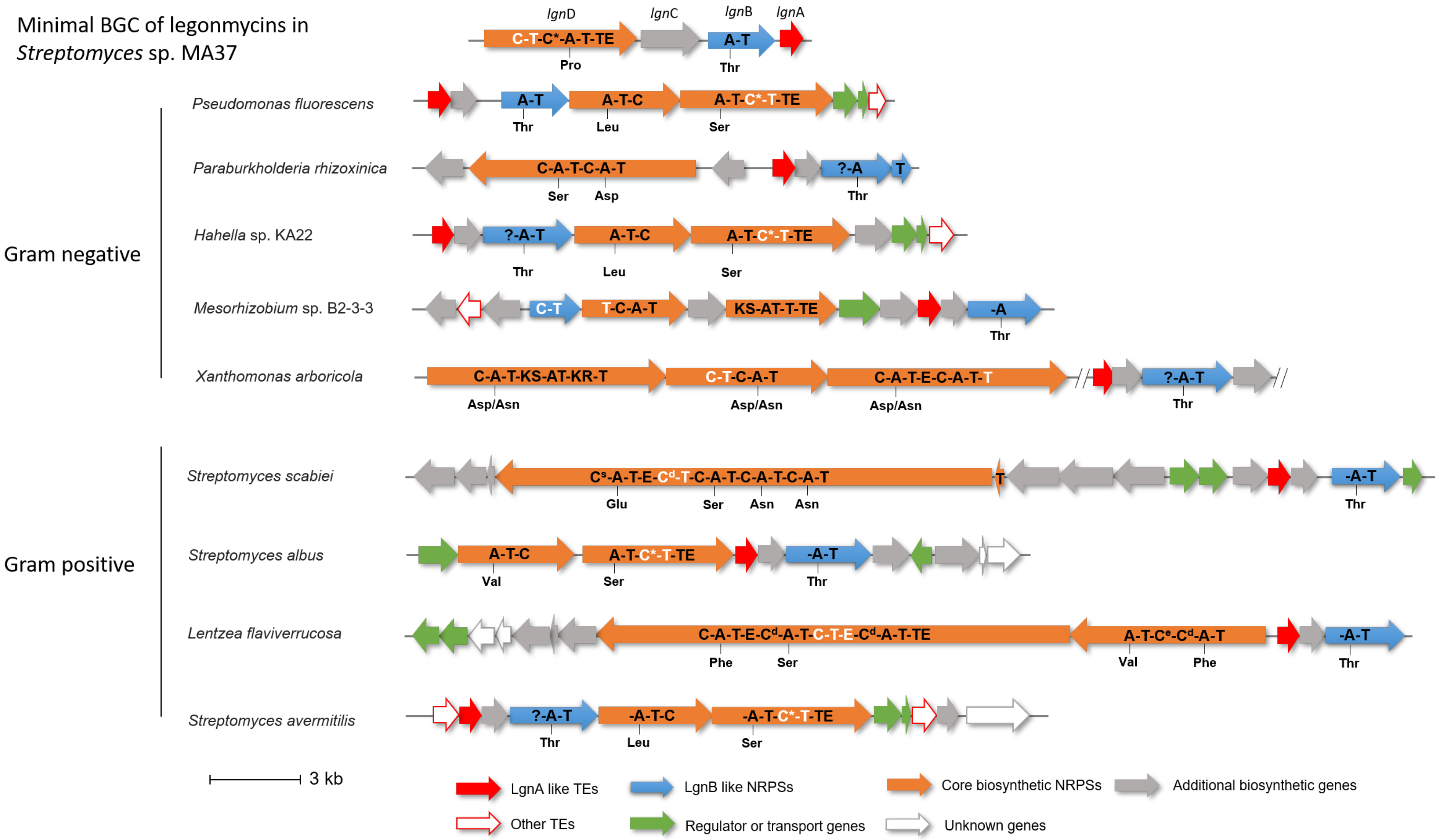

Supplementary Fig. 34. Representative BGCs containing LgnA-like TE domains identified by NCBI BlastP in comparison with the minimal BGC of legonmycins in *Streptomyces* sp. MA37 (top). The architectures of NRPS domains (i.e. C, A, TE, E domains) and the specificities of A domains are predicted using antiSMASH[4] and PKS/NRPS Analysis Website[5]. The specificities of C domains used were analyzed in NaPDoS database. [2] The extra C-T or T-C domains in the mega NRPS complexes are highlighted in white colour. C^d: DLC domain; C^E: epimerization domain; C*: unknown C domain.

Supplementary References:

1. Agarwal V, *et al.* Chemoenzymatic Synthesis of Acyl Coenzyme A Substrates Enables in Situ Labeling of Small Molecules and Proteins. *Org Lett*. **17**, 4452-5 (2015)

2. Ziemert, N. *et al.* The natural product domain seeker NaPDoS: A phylogeny based bioinformatic tool to classify secondary metabolite gene diversity. *PLoS One* **7**, 1–9 (2012).

3. Letunic, I. & Bork, P. Interactive Tree Of Life (iTOL) v4: recent updates and new developments. *Nucleic Acids Res.* **47**, W256–W259 (2019).

4. Blin, K. *et al.* antiSMASH 5.0: updates to the secondary metabolite genome mining pipeline. *Nucleic Acids Res.* **47**, W81–W87 (2019).

5. Bachmann, B. O. & Ravel, J. Methods for in silico prediction of microbial polyketide and nonribosomal peptide biosynthetic pathways from DNA sequence data. *Methods Enzymol.* **458**, 181–217 (2009).

6 Solovyev, V. & Salamov A. Automatic Annotation of Microbial Genomes and Metagenomic Sequences. in *Metagenomics and its Applications in Agriculture*, *Biomedicine and Environmental Studies* (ed. Li, R. W.) 61-78 (Nova Science 2011).
